# Identification of Potential Antiviral Inhibitors from Hydroxychloroquine and 1,2,4,5-Tetraoxanes Analogues and Investigation of the Mechanism of Action in SARS-CoV-2

**DOI:** 10.3390/ijms23031781

**Published:** 2022-02-04

**Authors:** Ryan S. Ramos, Rosivaldo S. Borges, João S. N. de Souza, Inana F. Araujo, Mariana H. Chaves, Cleydson B. R. Santos

**Affiliations:** 1 Graduate Program in Biotechnology and Biodiversity-Network BIONORTE, Federal University of Amapá, Macapá 68903-419, AP, Brazil; 2 Laboratory of Modeling and Computational Chemistry, Department of Biological and Health Sciences, Federal University of Amapá, Macapá 68902-280, AP, Brazil; lqfmed@gmail.com (R.S.B.); inana@unifap.br (I.F.A.); 3 Graduate Program on Medicinal Chemistry and Molecular Modeling, Institute of Health Science, Federal University of Pará, Belém 66075-110, PA, Brazil; 4 Chemistry Department, Federal University of Piauí, Teresina 64049-550, PI, Brazil; sammynery@ufpi.edu.br (J.S.N.d.S.); mariana@ufpi.edu.br (M.H.C.); 5 Binational Campus, Federal University of Amapá, Oiapoque 68980-000, AP, Brazil

**Keywords:** COVID-19, antiviral, receptor-binding domain

## Abstract

This study aimed to identify potential inhibitors and investigate the mechanism of action on SARS-CoV-2 ACE2 receptors using a molecular modeling study and theoretical determination of biological activity. Hydroxychloroquine was used as a pivot structure and antimalarial analogues of 1,2,4,5 tetraoxanes were used for the construction and evaluation of pharmacophoric models. The pharmacophore-based virtual screening was performed on the Molport^®^ database (~7.9 million compounds) and obtained 313 structures. Additionally, a pharmacokinetic study was developed, obtaining 174 structures with 99% confidence for human intestinal absorption and penetration into the blood–brain barrier (BBB); posteriorly, a study of toxicological properties was realized. Toxicological predictions showed that the selected molecules do not present a risk of hepatotoxicity, carcinogenicity, mutagenicity, and skin irritation. Only 54 structures were selected for molecular docking studies, and five structures showed binding affinity (ΔG) values satisfactory for ACE2 receptors (PDB 6M0J), in which the molecule MolPort-007-913-111 had the best ΔG value of −8.540 Kcal/mol, followed by MolPort-002-693-933 with ΔG = −8.440 Kcal/mol. Theoretical determination of biological activity was realized for 54 structures, and five molecules showed potential protease inhibitors. Additionally, we investigated the Mpro receptor (6M0K) for the five structures via molecular docking, and we confirmed the possible interaction with the target. In parallel, we selected the TopsHits 9 with antiviral potential that evaluated synthetic accessibility for future synthesis studies and in vivo and in vitro tests.

## 1. Introduction

An epidemic began in December 2019 in Wuhan, China, in which infected people suffered from pneumonia-like symptoms, which later spread throughout the world. The main cause of the infection was found to be a new virus that has structural similarities with coronaviruses related to severe acute respiratory syndrome, therefore called SARS-CoV-2 [1,2].

According to WHO data (https://www.who.int/emergencies/diseases/novel-coronavirus-2019/situation-reports) (accessed on 28 January 2022) globally, the number of new COVID-19 cases increased in the past week (17–23 January 2022) by 5%, while the number of new deaths remained similar to those reported during the previous week. Across the six WHO regions, over 21 million new cases were reported this week, representing the highest number of weekly cases recorded since the beginning of the pandemic. Nearly 50,000 new deaths were also reported. As of 23 January 2022, over 346 million confirmed cases and over 5.5 million deaths were reported worldwide. The current global epidemiology of SARS-CoV-2 is characterized by the dominance of the Omicron variant on a global scale, continued decline in the prevalence of the Delta variant, and very low-level circulation of Alpha, Beta, and Gamma variants. The Omicron variant includes Pango lineages B.1.1.529, BA.1, BA.2 and BA.3. BA.1, although several countries have reported recent increases in the proportion of BA.2 sequences. Preliminary evidence suggests there may be an increased risk of reinfection with Omicron, as compared to other variants of concern, but information is limited [3].

Due to the urgency of effective treatment strategies, the use of viral drugs is reported in the literature because they have great advantages, as the pharmacokinetics, pharmacodynamics, and safety profiles of these drugs are already well established [4,5,6]. Drug repositioning and ligand-based virtual screening have gained significant importance, as faster results and less investment are expected to identify a potent antiviral agent.

Preliminary studies have revealed lopinavir/ritonavir combination therapy as a potential inhibitor of the virus. Along with these two drugs, many other antiviral drugs have also been tested [7,8]. Recently, it was reported that the antimalarial drugs chloroquine and hydroxychloroquine have a certain curative effect on COVID-19; however, the drugs are alert to hepatotoxicity [9].

SARS-CoV-2 is a positive-sense, single-stranded RNA virus that relies on its Spike (S) protein to bind and enter target cells [10,11]. Virus protein S binds to the host cell’s Angiotensin-Converting Enzyme 2 (ACE2) receptor, allowing virus particles to enter cells. Thus, blocking the ACE2 receptor reveals a potential therapeutic target for drug discovery to prevent the transmissibility of SARS-CoV-2 [12]. Considering that the receptor-binding domain (RBD) is the important region for receptor interaction, antibodies that target conserved epitopes in the RBD also have great potential for the development of highly potent cross-reactive therapeutic agents against the SARS-CoV-2 [13].

The genomic RNA of coronaviruses is approximately 30,000 nucleotides long with a 5’cap structure and a 3’poly(A) tail and contains at least six open reading frames [14]. These polyproteins are processed by a major protease, Mpro (also known as a 3C-like protease (3CLpro)), and by one or two papain-like proteases, into 16 nonstructural proteins [15]. Therefore, these proteases, especially Mpro, play a vital role in the life cycle of coronaviruses. Mpro is a three-domain cysteine protease involved in most maturation cleavage events within the precursor polyprotein [16].

**Figure 1:**
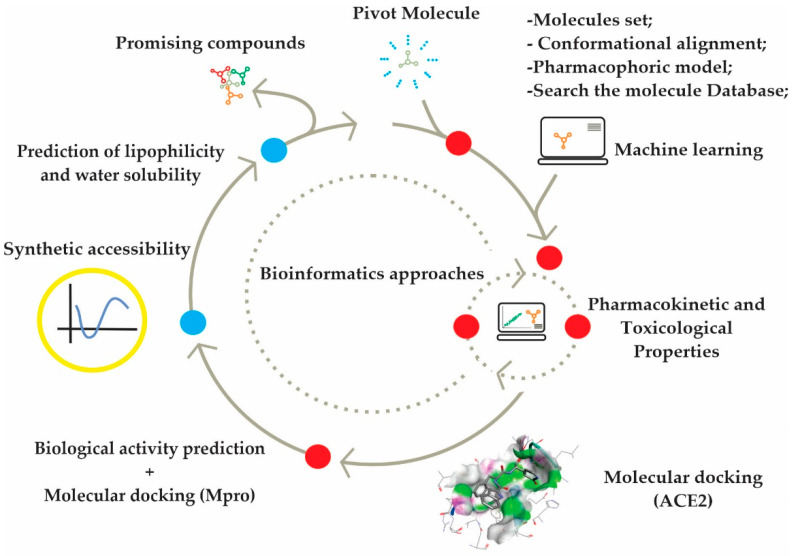
Flowchart of the methodological stages of the research.

The modeling of bioactive molecules is widely used in the identification of new prototypes with biological activity, and this search consists of pre-selecting compounds with the help of a computer from virtual databases [17]. A computer-aided drug design project saves costs and labor to test all compounds in the laboratory and helps to screen for potent ligands/inhibitors that can target most strains [18]. Therefore, this study aims to identify potential inhibitors and investigate the mechanism of action on the SARS-CoV-2 ACE2 receptor and main protease (Mpro), using the study of molecular docking from 1,2,4,5-tetraoxanes analogues. Figure 1 show the schematic flowchart of the methodological steps that were carried out in the research, joining efforts and different expertise in bioinformatics, and allowing the association necessary to achieve the proposed objectives.

## 2. Results and Discussion

### 2.1. Molecular Docking for Obtaining and Evaluating the Pose of Selected Structures and the Pharmacophoric Model

Hydroxychloroquine (HCQ) was used as a pivot molecule, according to Wang et al. (2020). HCQ bind to ACE2 with K_D_ = (7.31 ± 0.62) × 10^−7^ M and exhibit equivalent suppression effect for the entrance of 2019-nCoV spike pseudotyped virus into ACE2 cells [19]. In vitro studies reported that HCQ was effective against SARS-CoV-2 at a Multiplicity of Infection (MOI) of 0.01 with a 50% effective concentration (EC_50_) of 4.51 μM in Vero E6 cells. All MOIs (0.01, 0.02, 0.2, and 0.8) and EC_50_ for HCQ (4.51, 4.06, 17.31, and 12.96 μM) was satisfactory [20]. Thus, a molecule set of antimalarial analogs of 1,2,4,5 tetraoxanes and hydroxychloroquine as a pivot was preliminarily evaluated in the molecular docking study for the ACE2 target to evaluate the binding affinity and subsequent obtainment of the pharmacophoric model. The predicted inhibitory constant (pKi) was calculated using the following Equation (1) [21]:

(1) $$\mathrm{pKi}=10^{\;(\Delta G/1.366)}$$

Table 1 show the binding affinity values of the selected structures (see Supplementary Material—Figure S1) as well as the pharmacophoric characteristics that were obtained via the PharmaGist web server to obtain the pharmacophoric model, in which 14 selected molecules were used as an input file and hydroxychloroquine was added as a pivot structure with an alignment score of 65.383. Subsequently, a matrix was constructed (Table 1) with the following pharmacophoric descriptors: atoms (ATM), spatial features (FEA), hydrogen bond donor (DON), hydrogen bond acceptor (ACC) and binding affinity (BA).

Descriptors were analyzed using the Minitab^®^ v. 16 software, in which the most relevant ones were used to predict the potential antiviral activity as a function of the BA value to reduce statistical inconsistencies. The ACC showed a correlation of −0.870 (strong) compared to the other descriptors, which allows us to infer that the number of hydrogen donor groups significantly interferes in the BA responses of the selected molecules. However, the contribution of each descriptor in the process of potential antiviral activity is noteworthy, as is the case of ATM with a correlation value of −0.719, FEA of −0.769, HYD of −0.763, DON of 0.849, and ACC of −0.870, which also contributes to binding affinity.

**Figure 2:**
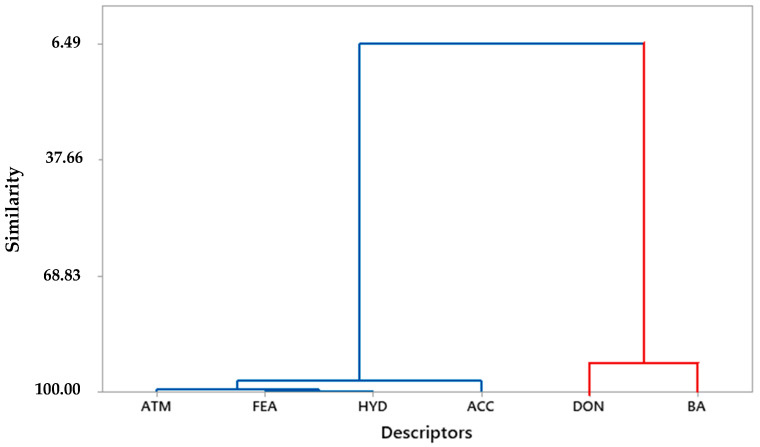
Dendrogram of hierarchical cluster analysis of pharmacophoric descriptors and binding affinity.

The descriptors with the strongest correlations (positive and negative) were selected for evaluation by chemometric study. Dendrograms were obtained from the HCA using Minitab^®^ software, see Figure 2. Confirmation of the data obtained by the Pearson correlation was facilitated by generating the pharmacophoric hypotheses of the HCA in which a correlation of binding affinity (BA) as an independent variable and the structural similarity cursor was performed in the categories: higher affinity (a) and lower affinity (b), from five molecular descriptors, atoms (ATM), Spatial Features (FEA), Hydrophobic (HYD), donor (DON), and hydrogen bond acceptor (ACC).

**Figure 3:**
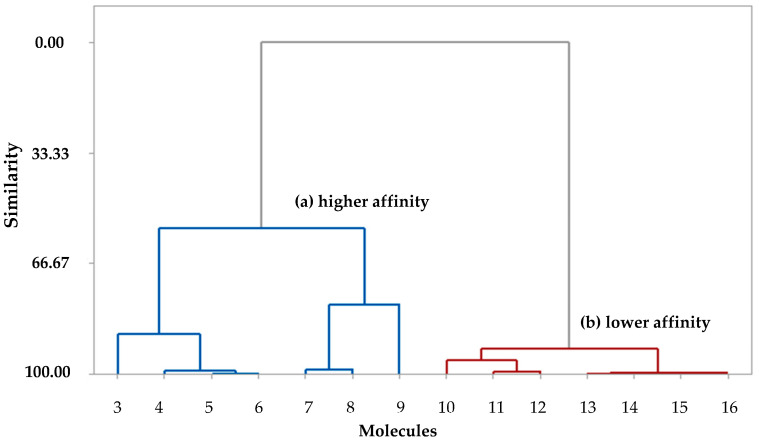
Dendrogram of HCA classifying structures with higher affinity (blue) and lower affinity(red).

The statistical analysis used in this study grouped structures of similar molecules into categories (Cluster). Categories are represented by a two-dimensional (2D) diagram known as a dendrogram. Molecules are represented by the branches at the bottom of the dendrogram. The similarity between clusters is given by the length of their branches so that compounds with low similarity have long branches while compounds with high similarity have short branches. The HCA method classified the molecules into two classes (high and low binding affinity) and was based on the Euclidean distance and the incremental method with full linkage [22]. HCA technique presented a similarity dendrogram in which the molecules were classified into two classes (with higher and lower binding affinity), according to their similarities, as shown in Figure 3.

The classification in the clusters considered the structural similarity in relation to the descriptors with the highest correlations. The binding affinity property stands out, which allows us to evaluate the possible interaction in the binding site, as significant values of higher BA (blue) of the structures were observed at 3–9. The cluster (blue) presents the molecules with the highest binding affinity values, consisting of molecules 3, 4, 5, 6, 7, 8, and 9. The cluster (red) classified the molecules with the lowest binding affinity value for structures 10, 11, 13, 14, 15, and 16.

Pharmacophore characteristics are essential when compared to the central molecule of the process, which has three HYD groups and three ACC groups, allowing the tracking of molecules with physical and chemical characteristics closer to those of hydroxychloroquine (Table 2).

Tophit 313 molecules were selected using the physicochemical descriptors tracking filter on the Pharmit web server (Table 3). The web server, in addition to the pharmacophore model, enables the special tracking filter when the stereochemical and electronic characteristics are simple and common, which allows for a more precise search with structural diversity for the construction of a small database of chemical structures.

The success of molecule virtual screening shows that potential biological activity depends on the precision and specificity of the activated pharmacophore [22]. Virtual screening through a database consisting of commercial molecules from Molport and internal molecules (real/virtual files extended from the real scaffold) as an internal 3D database prepared for the virtual tracking of any model [23]. Thus, a molecular fit was applied to the molecules selected from the virtual screening based on the pharmacophore model.

**Figure 4:**
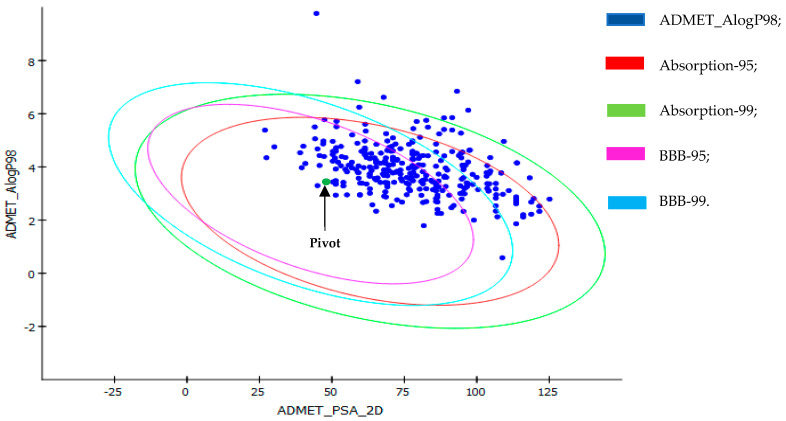
Polar surface area (PSA) versus ALogP plot showing molecules in the 95% and 99% confidence limit ellipses corresponding to the blood–brain barrier (BBB) and intestinal absorption (IA). Pivot-Hydroxychloroquine.

The 313 molecules obtained from the rapid tracking of pharmacophoric characteristics and the application of reduction filters were subjected to the prediction of pharmacokinetic properties. The plot of polar surface area and ALogP of the molecules is shown in Figure 4.

The screening of the molecule’s results in the DS-ADME model showed that of the 313 molecules subjected to pharmacokinetic prediction, only 182 have 99% confidence levels for human intestinal absorption and penetration into the blood–brain barrier (BBB). The other molecules are outside the ellipse filter of the ADME model, which indicates their lower intestinal absorption and low BBB penetration capacity. These ellipses define regions where well-absorbed molecules are expected to be found.

The compounds’ good absorption or permeation through the blood–brain barrier is measured by its LogP that must be less than five [23]. Results of pharmacokinetic screening revealed that 174 molecules followed Lipinski’s rule of five for oral bioavailability. Eight (8) molecule structures are out of the ellipse models because they show lipophilic nature due to the high LogP value, and of these, only one structure showed high lipophilicity and low membrane permeability due to the high LogP and molecular weight.

ADME descriptors of the molecules were calculated for drug similarity studies. Intestinal absorption and blood–brain barrier penetration were predicted by developing an ADME model using the 2D PSA and AlogP98 descriptors that include 95% and 99% confidence ellipses [24,25].

Considering the established absorption, distribution, metabolism, and excretion reference parameters [26] and the pivot, the molecules selected in the previous step were evaluated within these criteria, and 182 were selected, in which they satisfy the conditions [27,28,29]. Chemical structures with less, or preferably without, violations of these rules are more likely to be administered/available orally. The results of the pharmacokinetic prediction revealed that the most active structures followed Lipinski’s rule of five (R5) for oral bioavailability. The established reference ADME and pivot molecule parameters can be seen in Table 4 and Table 5; see Tables S1–S3 in Supplementary Material for extended information.

All molecules tested in the present study exhibit hydrogen bonding and hydrophobic interactions with corresponding amino acids, according to molecular docking simulations. The pivot structure did not present violation within the reference parameters (Lipinski’s rule), and this same condition was observed for all molecules, which can be exemplified by the great similarity between the tested molecules, thus corroborating the studies carried out. The USFDA (Food and Drug Administration) standard toxicity risk predictor software TOPKAT (Discovery Studio, Accelrys) locates fragments within the molecule structure that indicates a potential threat to toxicity risk [30]. Toxicological predictions results for the TopHits 9 molecules can be seen in Table 6 and Table 7; see Table S4 in Supplementary Material for extended information.

TOPKAT toxicity screening results for the selected compounds showed that the studied compounds do not present a risk of carcinogenicity, mutagenicity, and skin irritation, nor of skin sensitization capacity.

Similarly, the results of the USFDA rodent carcinogenicity toxicity screening, Ames mutagenicity, were negative; the test is used globally as an initial screening method to determine the mutagenic potential of new chemicals and drugs. In all parameters, ADMET and toxicological, the selected compounds (8) indicate values and characteristics superior to those of the pivot compound. Only the molecule Molport-009-499-144 showed a similar alert to hydroxychloroquine for the Ames mutagenicity test, requiring an investigation and in silico evaluation of the prediction of tolerated dose in an animal model. Molecules Molport-005-028-274, Mol-port-009-913-111, and Molport-002-693-933 present a mild prediction for skin irritation.

TOPKAT toxicity screening results for the TopHits 9 showed that the molecules studied do not present a risk of carcinogenicity, mutagenicity, and skin irritation; however, the warning can generate complications when compared to commercial compounds (Hydroxychloroquine) in development and reproduction if ingested at high doses or long-term therapeutic use in humans (see Table 7).

The carcinogenic potency data show that the molecules and pivot were within the maximum tolerated dose for rats which caused the mortality of 50% of the investigated population (TD_50_). The Molport-005-083-430 molecule, possessed a higher value of TD_50_; however, in a mouse model, had values within the tolerated dose (see Table 8).

### 2.2. Molecular Docking for ACE2 Receptor

**Figure 5:**
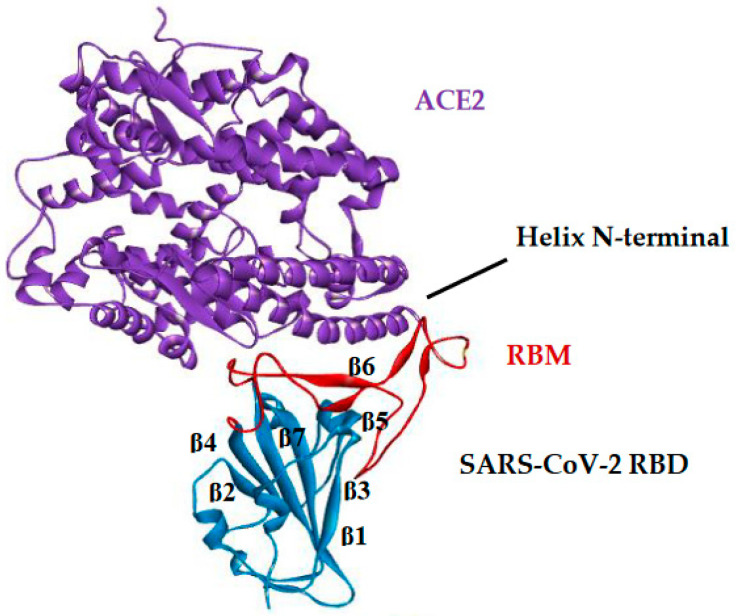
Major constituent components of SARS-CoV-2 RBD bound to ACE2.

The cryo-electron microscopy structure of the SARS-CoV-2 Spike trimer was recently reported in two independent studies. However, an inspection of the available spike protein structure revealed incomplete modeling of the RBD, particularly for the Receptor Binding Motif (RBM) that directly interacts with ACE2 [34,35]. The general structure of SARS-CoV-2 RBD together with its subunits and constituent parts, can be seen in Figure 5.

SARS-CoV-2 RBD has five antiparallel β-sheet twisted strands (β1, β2, β3, β4, and β7) with short connecting helices and loops that form the nucleus [35]. Between the β4 and β7 strands in the core, there is an extended insert containing the short β5 and β6 strands, α4 and α5 helices, and loops (see Figure 5).

**Figure 6:**
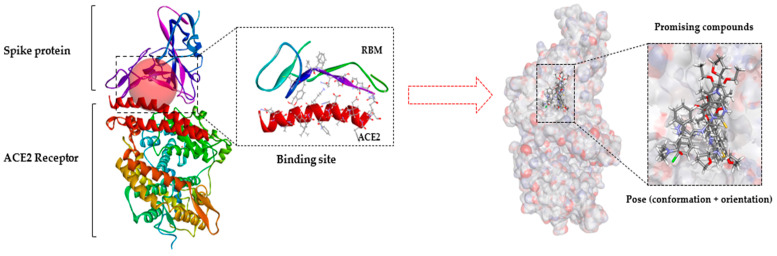
General structure of SARS-CoV-2 RBD binding to ACE2 molecular docking of promising compounds.

Given the large contact surface between Spike’s RDB domain and ACE2, to carry out docking studies at this binding site, the grid configuration was centered on the Cα of the Gln493 residue located at the interface of the interaction between Spike and ACE-2, as shown in Figure 6.

According to Lan et al. (2020) [26], in vitro binding measurements showed that SARS-CoV-2 RBD binds to ACE2 in a low-affinity range (nanomolar), indicating that RBD is a key functional component within the subunit S1 which is responsible for binding SARS-CoV-2 on ACE2. In comparison, in alignment and mapping studies in their respective sequences, the residues interact with ACE2 in the RBDs SARS-CoV-2 and SARS-CoV.

It is worth highlighting that there are 14 shared amino acid positions used by both RBMs for the interaction with ACE2 and 8 have identical residues between the two RBDs, including Tyr449/Tyr436, Tyr453/Tyr440, Asn487/Asn473, Tyr489/Tyr475, Gly496/Gly482, Thr500/Thr486, Gly502/Gly488, and Tyr505/Tyr491 from SARS-CoV-2/SARS-CoV, respectively. Five positions have residues that have similar biochemical properties despite having different side chains, including Leu455/Tyr442, Phe456/Leu443, Phe486/Leu472, Gln493/Asn479, and Asn501/Thr487 of SARS-CoV-2/SARS-CoV, respectively.

This extended insert is the RBM, which contains most of the SARS-CoV-2 contact residues that bind to ACE2 [36,37]. The N-terminal peptidase domain of ACE2 has two lobes, forming the peptide substrate binding site between them.

**Figure 7:**
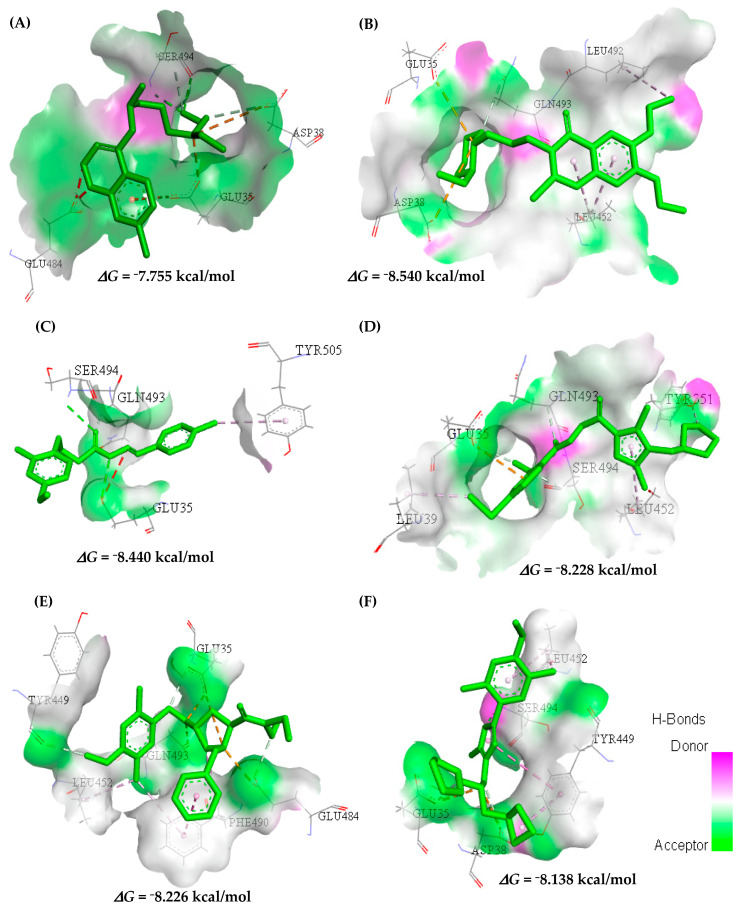
Interactions of the ligands (**A**) Hydroxychloroquine, (**B**) MolPort-007-913-111, (**C**) MolPort-002-693-933, (**D**) MolPort-004-042-669, (**E**) MolPort-005 -131-430, and (**F**) MolPort-005-060-605 in the Spike RBD active site. Ligands are shown as green rods, Spike RBD residues are shown as atom-like colored rods, hydrogen bonds formed between the ligands and the receptor are represented as green dotted lines, π–π type interaction as lines yellow dotted lines and π–cation interaction as red dotted lines.

The docking poses of all the main molecules show that they interact in a conformation that fits them into the binding pocket of the RBM. The docking poses, along with their respective interactions, are shown in Figure 7.

The generated docking poses made it possible to observe that the ligands interact with the amino acid residues of the active site of Spike RBD (PDB ID 6M0J) around the α-helix between the Tyr449-Tyr505 amino acid residues and comprised in the β-sheet between the residues of Glu35-Asp39 amino acids. In ligands, it is possible to observe hydrophobic interactions with many residues in Leu39, Tyr449, Leu452, Phe490, and Leu492; these results agree with studies in the literature [38].

In the study of molecular docking, the interactions of potential inhibitors with the amino acid residues Tyr449, Gln493, Ser494, and Tyr505 in Spike RBD are similar to those reported in the literature [39,40,41]. The best-evaluated inhibitors in terms of binding affinity were (B) MolPort-007-913-111 (−8.540 kcal/mol) and (C) MolPort-002-693-933 (−8.440 kcal/mol), in the which interactions were like those observed in the control for residues Glu35 and Ser494, contributing to the increase in binding affinity. The unusual interactions between the inhibitors were Leu39, Tyr351, Tyr 449, Phe490, Glu494, and Tyr505, and these contributions help to stabilize the active site for Spike inactivation in the RBM domain, see Figure 7.

**Figure 8:**
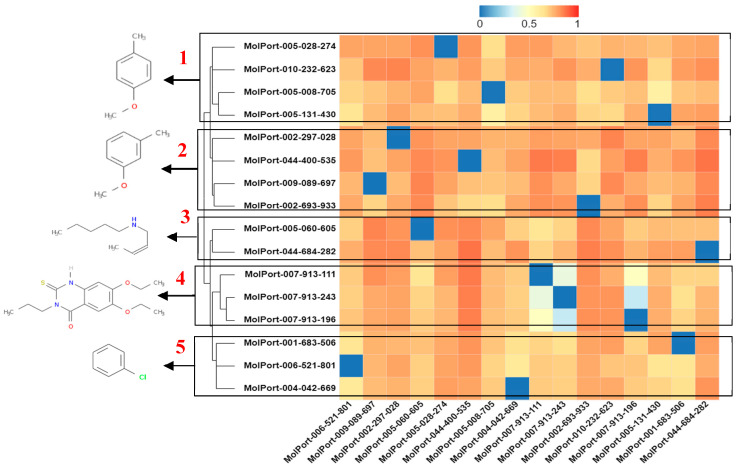
Heatmap of hierarchical cluster analysis based on the Tanimoto index of selected molecules for ACE2.

A heatmap of the hierarchical cluster analysis of molecules can be seen in Figure 8. The analysis was performed to select the molecule with the highest representation from each group based on structural dissimilarity. It is known that similar molecules have a similar mechanism of action [42], as there is still no known drug for the treatment of SARS-CoV-2 (ACE2 target).

In the heatmap of the molecules selected in the ACE2 target, cluster 1, the molecule that presents a chemical structure profile with greater dissimilarity from the others is MolPort-005-131-430, with a Tanimoto index (IT) of 0.23 for the 1-methoxy -4methylbenzene fragment. The interaction of the Tyr449 residue (hydrogen bonding) with the fragment stands out in the binding affinity value (molecular docking study) for the molecules of the group. In cluster 2, the molecule MolPort-002-693-933 is observed, which presents IT of 0.31 for the 1-methoxy-3methylbenzene fragment and stands out in the binding affinity value compared to the others belonging to the group, see Figure 8.

The *N*-[(2Z)-but-2-en-1-yl]pentan-1-amine fragment with IT of 0.27 is found in the molecule MolPort-005-060-605 in cluster 3, the interactions (Glu35 hydrogen bonding, Asp38 and Ser494, hydrophobic Tyr449, Leu452 and related electrostatic Asp38) enable the expressive value of binding affinity. In cluster 4, a IT of 0.51 was seen for the 6,7-diethoxy-3-propyl-2-thioxo-2,3-dihydroquinazolin-4(1H)-one fragment of the MolPort-007-913-111 molecule with Pi-alkyl interactions and alkyl. In cluster 5, the benzene fragment with an IT of 0.33 from MolPort-004-042-669 has expressive Pi–anion interactions with the residue Glu35 and Pi-alkyl with Leu39.

### 2.3. In Silico Determination of Biological Activity and Molecular Docking Simulations (Mpro)

In a study by Refaey et al. (2021) [43] regarding repositioning renin inhibitors as SARS-CoV-2 main protease inhibitors, five pharmacophoric characteristics were found in the pharmacophoric model, constituting two hydrogen acceptor and three hydrophobic groups, thus, these results corroborate the data obtained in this research.

Therefore, in this study, we realize the theoretical determination of biological activity for 54 structures, and only five molecules showed the potential of protease inhibitors, see Table 9 (Tophits 5 and control). The control compound (11b, ~{N}-[(2~{S})-3-(3-fluorophenyl)-1-oxidanylidene-1-[[(2~{S})-1-oxidanylidene-3-[(3~{S})-2-oxidanylidenepyrrolidin-3-yl]propan-2-yl]amino]propan-2-yl]-1~{H}-indole-2-carboxamide) showed SARS-CoV-2 Mpro inhibitory potential, validating the results predicted in this study. The exploration of biological activities of the selected compounds through PASS analysis resulted in similar kinds of biological activities.

Compound MolPort-009-219-532 presented predictions to be considered both a protease and enzymatic inhibitor, see Table 9. MolPort-005 -028-274 has shown prediction protease inhibitors with a 0.134 probability to be active (Pa).

**Figure 9:**
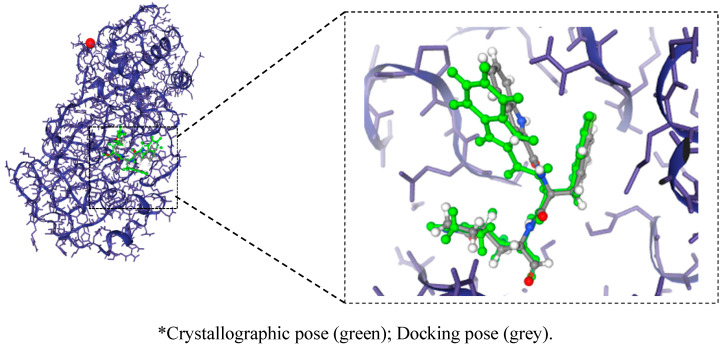
Validation of the molecular docking protocols for the crystal structure of the main protease (Mpro) COVID-19 in complex with 11b inhibitor *.

The molecular docking validation results were considered satisfactory, in which the relative positions of the crystallographic ligand and the coupled ligand were similar (Figure 9). The RMSD between the atoms of the crystallographic ligand (Mpro) and the coupled ligand was calculated to be 1.519 Å.

According to Gowtham et al. (2008) [44] and Hevener et al. (2009) [45], the predicted binding mode using molecular docking indicates that when the RMSD is less than 2.0 Å in relation to the crystallographic pose of a respective ligand, the validation is considered satisfactory [46,47].

The 3CLpro/MPro activity site is found in the gap between domains I and II, consisting of a Cys-His catalytic dyad (Cys145 and His41) [48]. The active pocket consists of hydrophobic amino acids such as Tyr54, Met49, Met165, Phe140, Leu141, Cys145, Leu27, Pro168, Leu167, Cys145, Ala191, Cys44, Leu50, and Met40, which provide a relatively hydrophobic environment to contain the compound and stabilize its conformation.

**Figure 10:**
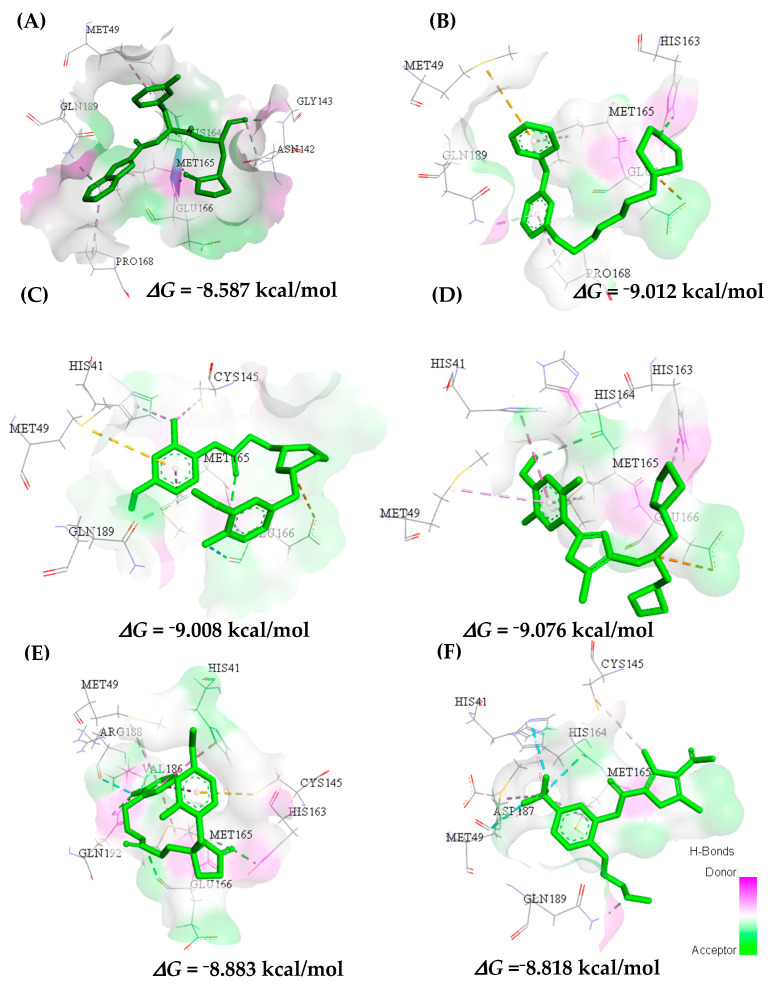
Ligand interactions (**A**) 11b, (**B**) MolPort-009-219-532, (**C**) MolPort-004-996-519, (**D**) MolPort-005-060-605, (**E**) MolPort-005 -028-274, and (**F**) MolPort-009-499-144 in the active site of Mpro.

In control 11b (A) the interactions observed in the docking study were also similar in molecules (B) MolPort-009-219-532 and (D) MolPort-005-060-605 in relation to the active site, and others not common in molecules (C) MolPort-004-996-519, (E) MolPort-005-028-274, and (F) MolPort-009-499-144. The interactions are located around the α-helix between the amino acid residues Met49-Glu47 and in the β-sheet at residues His163-164 and Glu166, as shown in Figure 10.

The selected molecules were docking with binding affinity energies to the Mpro target, and thus, a range of −8.587 to −9.012 kcal/mol was observed. The values found for the binding affinity in the docking study of all molecules are shown in the Supplementary Material, see Table S5. In the selected chemical structures, only molecule (B), MolPort-009-219-532, showed superior and/or similar results for binding affinity (−9.012 kcal/mol) compared to the values of the controls used in the study of molecular docking (11b: −8.587 kcal/mol; Lopinavir: −9.680 kcal/mol and Ritonavir: −9.594 kcal/mol). In (B), MolPort-009-219-532 interactions with hydrogen bonds and amino acid residues His163 and Gln189 were observed, suggesting the stabilization of the formed complex and the contribution of hydrophobic interactions with residues Met165-Pro168; Pi-sulfur Met49 and electrostatic interactions withthe Glu166 residue were also seen. The other molecules used in the molecular docking study, despite showing a lower binding affinity value, showed interactions similar to those observed for control groups and were not common among residues Gln189, Gln192, Asp187 and His164.

Around the α-helix (Glu47-Leu50) of the crystallographic structure, Mpro has a conformation that allows interaction with the Met49 residue, while those located in the β-sheet bind to the His163-Glu166 amino acids on the active sites of the receptor (PDB 6M0K). The major interactions involved were of the conventional hydrogen bond type observed in residues His163 and Glu166, located in the β-sheet, and Gly143 in the loop of the macromolecule. In the α-helix, Met49, Met165, and Pro168 residues appear in processes involved in hydrophobic electronic interactions, respectively. Therefore, this study showed that the results agree with studies in the literature [49,50].

**Figure 11:**
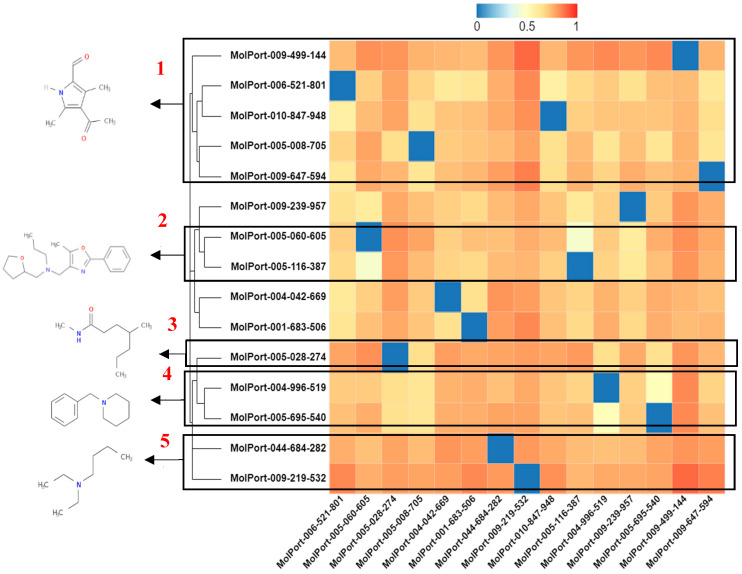
Heatmap of hierarchical cluster analysis based on the Tanimoto index of selected molecules for Mpro.

In the heatmap (Figure 11) of selected molecules in the Mpro target in cluster 1, the 4-acetyl-3,5-dimethyl-1H-pyrrole-2-carbaldehyde fragment with an IT of 0.26 (MolPort-009-499-144) has an expressive hydrophobic interaction with the Cys145 residue. In cluster 2, the *N*-[(5-methyl-2-phenyl-1,3-oxazol-4-yl)methyl]-*N*-(tetrahydrofuran-2-ylmethyl)propan-1-amine fragment of the MolPort-005-060-605 molecule with an IT of 0.54 has hydrophobic interactions with His41, Met49, and Met165 residues and hydrogen bonding with His164.

In cluster 3, the N,4-dimethyl heptanamide fragment of the MolPort-005-028-274 molecule with an IT of 0.32 has conventional hydrogen bond interactions with the Glu 166 residue. In cluster 4, the 1-benzylpiperidine fragment from MolPort-004-996-519 with an IT of 0.486289 has electrostatic interactions with Glu166; and C-H binding with Gln189. At 5, the 1-benzylpiperidine fragment from MolPort-009-219-532 with an IT of 0.486289 shows electrostatic interactions with the Glu166 residue. The dissimilarity analysis allowed us to observe the contribution of each fragment to the possible interactions at the site of activity in the molecular targets of the study.

### 2.4. Synthetic Accessibility (SA) Prediction

The molecules selected in the molecular docking study were subjected to synthetic accessibility (SA) prediction and presented chemical accessibility predicted as easy, obtaining a score above 60 for both ACE2 and Mpro; that is, the molecules are easily synthesized, as shown in Table 10, see Table S6 in the Supplementary Material for extended information.

**Figure 12:**
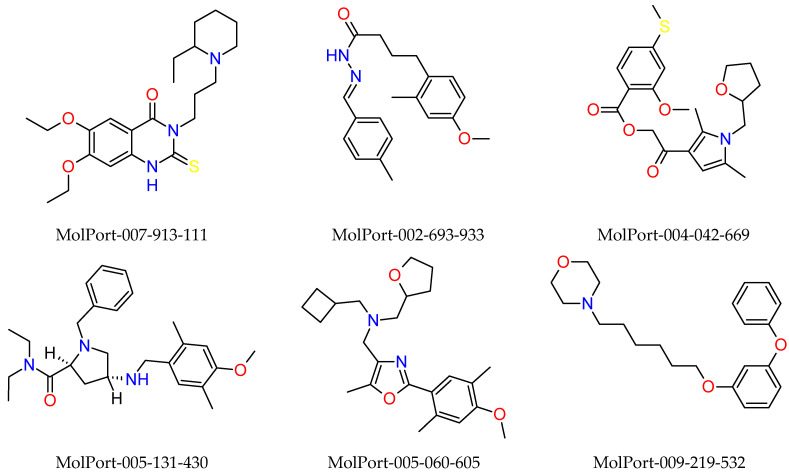

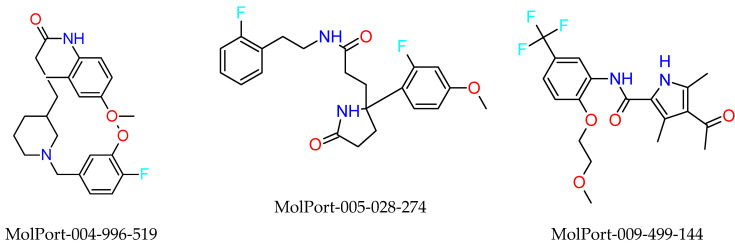
Molecules selected in the in silico study.

Synthetic accessibility was obtained by the AMBIT web server (ambit.sourceforge.net/reactor.html) (accessed on 31 May 2021) [51]. AMBIT calculates the complexity parameters of a molecule and issues a score ranging from 0 to 100, where 100 is the accessibility value synthetic maximum (easy synthesis) and 0 is the minimum (greater difficulty of synthesis) [52]. At the end of the virtual screening stages, nine molecules presented a better profile for SARS-CoV-2 inhibitory potential, according to Figure 12.

### 2.5. Prediction of Lipophilicity and Water Solubility for Promising Compounds

A parameter, commonly logP, is used to express the liposolubility of drugs, and it becomes the key point for drug planning [53]. This property affects the ability of a molecule to decompose and to decompose in non-polar environments versus aqueous environments. The nine promising compounds showed consensus logP values spanning from 3.54 to 4.26, see Table 11.

In fact, in this study, only positive logP values in the range of 1.56 to 5.71 were found. It is worth mentioning that such positive values indicate that all molecules that are highly lipophilic meet an essential criterion for a drug candidate [54].

The promising compounds showed consensus regarding logS values in the range of −4.64 to −6.26, as shown in Table 12. In this study, only negative logS values in the range −3.91 to −8.57 were found. A logS reference value for moderate solubility is between −4 and −6, −2 to −4 indicates good solubility and values greater than −6 indicate poor solubility. Solubility in water is an important requirement for any drug candidate molecule, considering its oral or parental administration, as there are many active pharmaceutical ingredients that must be administered in small volumes [55]. Therefore, we can conclude that the pivot molecule and nine promising compounds are moderately soluble in water, and the compound MolPort-005-028-274 is poorly soluble in water.

## 3. Materials and Methods

### 3.1. Obtaining, Optimizing, and Molecular Docking for Selected Structures

Initially, hydroxicloquine, chloroquine, and 14 structures of 1,2,4,5 tetraoxane analogues were selected from the literature with proven in vitro testing against malaria caused by *Plasmodium falciparum*—Sierra Leone clone D-6 to form the training set (Figure S1, see Supplementary Materials), followed by chemometric analysis studies [56]. The molecules were optimized by the computational method DFT B3LYP 6-31G** to obtain bioactive conformation and later used as input files (in .mol and .sdf formats). Hydroxychloroquine was used as a control molecule because it has selective antimalarial activity, and the molecules were subjected to a molecular docking study in order to evaluate the binding affinity at the binding site in the receptor–binding domain in the Spike of SARS-CoV-2 linked to ACE2 with PDB ID 6M0J using the DockThor program in order to select the best bioactive pose (conformation + orientation) at the binding site for future analyses to obtain the pharmacophoric model. The methodological step can be consulted in more detail in Section 2.2 and Section 3.5.

### 3.2. Generation and Evaluation of the Pharmacophoric Model

The input file with the pivot and molecules with the best binding affinity values were submitted to the PharmaGist web server (https://bioinfo3d.cs.tau.ac.il/PharmaGist/) (accessed on 6 March 2021) to determine the pharmacophoric characteristics: Atoms (ATM), Spatial Features (SF), Features (F), Aromatic (ARO), Hydrophobic (HYD), Acceptors (ACC) Donors (DON) [57]. An alignment score was used to choose the model and was later evaluated by the incremental method via Hierarchical Cluster Analysis (HCA) and Pearson correlation (pharmacophoric characteristics with binding affinity of studied molecules).

### 3.3. Selection of Molecules in the Database

The pharmacophoric model with the best alignment score was submitted to the Pharmit web server (http://pharmit.csb.pitt.edu/search.html) (accessed on 6 March 2021) for selection of the Top2000 molecules in the MolPort^®^ database (~7.9 million compounds) (Riga, NY, USA), based on pharmacophoric characteristics and filter (maximum and minimum) values of molecular descriptors, to increase the structural diversity in the virtual strategy [58].

### 3.4. Prediction of Pharmacokinetic and Toxicological Properties

Calculations of predictions of absorption, distribution, metabolism, excretion, and toxicity (ADMET) were performed using Discovery Studio v16, San Diego, CA, USA (2013) software [59,60]. These properties are important in determining the compound’s success for human therapeutic use. Some important chemical descriptors correlate well with ADMET properties, such as Polar Surface Area (PSA) as a primary determinant of fraction absorption and low Molecular Weight (MW) for oral absorption. The distribution of compounds in the human body depends on factors such as the blood–brain barrier (Log BB), permeability such as Caco-2 apparent permeability, MDCK cell apparent permeability, Log Kp for skin permeability, the volume of distribution, and binding to plasma proteins (Log Khsa for protein binding).

Toxicity prediction tests were performed using Discovery Studio v.16 software via the Toxicity Prediction function by Komputer Assisted Technology (TOPKAT). Toxicity parameters included carcinogenicity in rodents, mutagenicity, the Ames test, skin irritation, eye irritation, aerobic biodegradability (AB), oral toxicity in rats (LD_50_ in g/kg body weight), and whether the molecule was non-carcinogenic, non-mutagenic or non-degradable.

### 3.5. Molecular Docking for ACE2 Receptor with DockThor

Correct assignment of protein and ligand protonation/tautomeric states is crucial to the binding mode and its affinity predictions, requiring careful inspection of the structures. In this research, the complexes were prepared using the PDB2QR web server (https://server.poisonboltzmann.org/pdb2pqr) (accessed on 6 March 2021) [61,62]. The assignment of protonation and tautomeric states of the ligands was performed with the Discovery Studio program, while the hydrogen atoms of the protein were added with PROPKA using pH 7.

The crystal structure of the Spike receptor binding domain of SARS-CoV-2 linked to ACE2 (Homo sapiens organism) with PDB ID 6M0J [63], resolution of 2.45 Å, and elucidated by the X-ray diffraction method.

The DockThor program uses a topology file for the ligand and cofactors (.top) and a protein-specific input file (.in) that contains the atom and partial charge types of the MMFF94 force field, both of which are generated using the built-in tools MMFFLigand and PdbThorBox. The PdbThorBox program is used to define the protein atom types and the partial charges of the MMFF94 force field. Thus, in the DockThor program, protein and ligands (including cofactor molecules) are treated with the same force field in the docking experiment [64].

The grid box configuration of each complex was automatically determined according to the reference binder when available: (1) The center of the coordinates was defined as the center of the coordinates of the ligand. (2) The grid size was defined as the largest value of the ligand axis but with a tolerance of 6 Å in each dimension. (3) Discretization (i.e., spacing between grid box points) was set to the default value of 0.25 Å.

The default parameters of the algorithm were defined as follows: (1) 24 docking runs, (2) 1,000,000 evaluations per docking run, and (3) population of 1000 individuals [65]. The quality of the protein–ligand docking score was evaluated based on the Root Mean Square Deviation (RMSD) between the best score of the docking pose and the experimental binding mode of the crystal ligand. The literature describes the common limit used to consider a highly flexible ligand coupling pose as an active type of conformation when the backbone RMSD value is ≤2.0 Å [46].

### 3.6. In Silico Determination of Biological Activity and Molecular Docking Simulations (Mpro)

Predictions of biological activity were performed using the online PASS web server, available at http://www.pharmaexpert.ru/passonline (accessed on 3 June 2021) [66]. Using PASS, it was possible to discover the effects of a compound based entirely on the molecular formula using MNA (multilevel neighbors of atoms) descriptors, suggesting that the biological activity is in the function of its chemical structure [67]. The drug-likeness calculations were carried out in Molinspiration analyses. Only molecules with protease inhibitors and enzyme inhibitors were selected at this stage.

#### Molecular Docking for Mpro Receptor

The crystal structure of the main protease (Mpro) of COVID-19 in a complex with the inhibitor 11b, PDB ID 6M0K [68] and a resolution of 1.50 Å were downloaded in the Protein Data Bank (PDB) in the format (.pdb) to perform an interaction study and receptor–ligand binding mode in the study of molecular docking. The hydroxychloroquine and 11b ligands were used as positive controls, and all water molecules and cofactors were deleted.

### 3.7. Structural Similarity and Synthetic Accessibility (SA) Prediction

Hierarchical clustering methods were used to select the molecules with the ChemMine Tools server, in which the measures of structural similarity of the clusters were calculated from atomic descriptors between each molecular pair, which generated a similarity matrix based on unique and common characteristics observed between molecules using the Tanimoto Index (0 = less similar and 1 = greater similarity). In the subsequent grouping steps, the similarity matrix was converted to a distance matrix by subtracting the similarity values from 1. The similarity search by ChemMine Tools allowed the structural comparison of ligands and their grouping according to similarity based on the Tanimoto Index [69].

The prediction of the synthetic accessibility (SA) of the molecules was performed using the AMBIT program (http://ambit.sourceforge.net/reactor.html) (accessed on 3 June 2021). The model for SA uses four weighted molecular descriptors, which represent different structural and topological features combined in an additive scheme [51,70]. In each target molecule or set of molecules, the algorithm calculates the molecular complexity; the stereochemical complexity is the complexity due to the presence of fused and bridged systems. The SA is issued as a score ranging from 0 to 100, where the value 100 is the maximum synthetic accessibility; that is, the molecule is more easily synthesized.

### 3.8. Prediction of Lipophilicity and Water Solubility for Promising Compounds

Promising molecules were evaluated with SwissADME software [53,55,56] for the prediction of lipophilicity and water solubility expressed by means of values of logP and logS, respectively. SwissADME provides five methods to predict logP values: iLOGP, xLOGP3, WLOGP, MLOGP, and Silicos-IT. iLOGP is an internal physical method of SwissADME, based on free solvation energies in 1-octanol and water calculated by the Generalized-Born model and access to surface area solvent (GB/SA). It has a performance equal to or greater than six well-established predictors.

## 4. Conclusions

The COVID-19 related pandemic is a fight that still needs to be fought by humanity, and beyond prevention by vaccination, the only way out is through the discovery of new drugs. Our study identified some potential candidates that can be used for the inhibition of Spike protein and Mpro in COVID-19.

ADMET studies have revealed that most molecules have good absorption properties and low acute toxicity values. Molecular docking studies confirmed the binding of molecules at the ACE2 active site, in which the molecule MolPort-007-913-111 had the best binding affinity value of −8.540 Kcal/mol, followed by MolPort-009-219-532 −9.012 Kcal/mol to Mpro. The similarity analysis was developed to guide future studies of molecular dynamics (MD) in the selection of these compounds. Promising compounds present binding affinity values with non-significant differences, and it may happen that when submitted to MD simulations, the behavior within the system is equivalent because they present great structural similarity. In this way, the structures of the compounds were evaluated based on the differences evident in each comparison cluster, both for the set of compounds selected in ACE2 and in Mpro. Additional experimental studies (in vitro and in vivo) need to be carried out to test possible candidates since they are easy to be synthesized, and thus better clarify the mechanism of action of the virus in the human organism.

## Figures and Tables

**Table 1:** Pharmacophoric model descriptors, binding affinity (BA), and results of the Pearson correlation matrix.

Molecules	ATM	FEA	HYD	DON	ACC	BA	pKi (µM)
1 *	49	10	3	2	3	−7.755	2.103
2	48	9	4	1	2	−7.709	2.273
3	96	34	24	0	10	−9.413	0.128
4	99	35	25	0	10	−9.230	0.175
5	105	37	27	0	10	−9.216	0.179
6	114	43	33	0	10	−9.214	0.180
7	102	37	27	0	10	−9.031	0.245
8	98	37	26	0	10	−9.009	0.254
9	101	38	27	0	10	−8.681	0.442
10	106	36	26	1	9	−8.655	0.461
11	103	37	27	1	9	−8.600	0.506
12	103	36	26	1	9	−8.589	0.516
13	106	36	26	1	9	−8.533	0.567
14	109	37	27	1	9	−8.533	0.570
15	103	37	27	1	9	−8.529	0.573
16	106	37	27	1	9	−8.526	0.607
**ATM**	1.000	-	-	-	-	-	-
**FEA**	0.988	1.000	-	-	-	-	-
**HYD**	0.989	0.998	1.000	-	-	-	-
**DON**	−0.477	−0.563	−0.563	1.000	-	-	-
**ACC**	0.939	0.967	0.955	−0.671	1.000	-	-
**BA**	−0.719	−0.769	−0.763	0.849	−0.870	1.000	-

* Pivot Molecule; Atoms (ATM), Features (FEA), Aromatic (ARO), Hydrophobic (HYD), Acceptors (ACC) Donors (DON).

**Table 2:** Characteristics obtained in the pharmacophoric model.

Pharmacophoric Characteristics		Coordinates			
		X	Y	Z	Radius (Å)
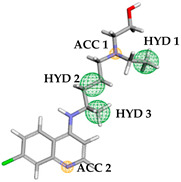	Hydrogen bond acceptor (ACC 1)	30.171	−13.304	−1.102	0.5
	Hydrogen bond acceptor (ACC 2)	26.428	−22.656	−0.807	0.5
	Hydrophobic (HYD 1)	32.525	−13.999	−1.149	1.0
	Hydrophobic (HYD 2)	28.372	−15.871	−0.992	1.0
	Hydrophobic (HYD 3)	28.789	−18.153	−2.161	1.0

**Table 3:** Application filter of the physicochemical descriptors of the selected molecules.

Molecules	MW	RotBonds	LogP	TPSA	ARO	HBA	HBD
1 *	335.88	9	4.00	48.38	2	4	2
2	319.87	8	5.00	28.16	2	2	1
3	620.36	9	7.45	115.85	0	10	0
4	634.37	9	7.69	115.85	0	10	0
5	662.40	9	7.93	115,85	0	10	0
6	704.45	8	7.38	126.84	0	10	0
7	648.39	9	7.63	126.84	0	10	0
8	633.36	9	6.91	126.84	0	10	0
9	647.38	8	6.63	132.64	0	9	1
10	661.42	10	7.88	118.64	0	9	1
11	647.40	8	6.77	118.64	0	9	1
12	647.40	9	7.15	118.64	0	9	1
13	661.42	10	7.65	118.64	0	9	1
14	675.44	8	6.40	132.64	0	9	1
15	647.40	8	6.77	118.64	0	9	1
16	661.42	9	7.15	118.64	0	9	1
Min.	319.872	8	4.00	28.16	0	2	0
Max.	704.450	10	7.93	132.64	2	10	2

* Pivot Molecule; MW: Molecular Weight; RotBond: rotative bonds; TPSA: Topological Polar Surface Area; Aro: Aromatic; HBA: Hydrogen Bond Acceptor; HBD: Hydrogen Bond Donnor.

**Table 4:** Prediction of pharmacokinetic properties for the molecules TopHits9.

Molecules	Oral Bioavailability	MW	AlogP	HBD	HBA	R5
Normal range	(<140 A°^2^)	(<500)	(≤5)	(≤5)	(≤10)	Max 4
Hydroxychloroquine	48.239	335.872	3.457	2	4	0
MolPort-009-219-532	30.142	355.471	4.755	0	4	0
MolPort-004-996-519	51.323	414.513	4.588	1	4	0
MolPort-005-060-605	45.027	398.538	4.677	0	4	0
MolPort-005-028-274	69.152	416.461	3.347	2	3	0
MolPort-004-042-669	66.740	417.518	3.416	0	6	0
MolPort-007-913-111	54.676	419.581	4.826	1	5	0
MolPort-002-693-933	50.364	324.417	4.586	1	3	0
MolPort-005-083-430	40.152	426.618	4.778	0	5	0
MolPort-009-499-144	80.327	398.376	3.166	2	4	0

Abbreviations: AlogP, the logarithm of the partition coefficient between n-octanol and water; MW: Molecular Weight; HBD: Hydrogen Bond Donnor; HBA: Hydrogen Bond Acceptor; R5: Lipinski Violations.

**Table 5:** Computational pharmacokinetic parameters (ADME) of TopHits9 structures.

Molecules	PPB	Hepatotoxic	CYP2D6	Solubility	BBB	IA
Hydroxychloroquine	false	true	true	3	1	0
MolPort-009-219-532	true	false	true	2	0	0
MolPort-004-996-519	true	false	false	2	1	0
MolPort-005-060-605	true	false	false	2	1	0
MolPort-005-028-274	true	false	false	2	2	0
MolPort-004-042-669	true	false	false	2	2	0
MolPort-007-913-111	false	false	false	2	1	0
MolPort-002-693-933	true	false	false	2	1	0
MolPort-005-083-430	true	false	true	2	1	0
MolPort-009-499-144	true	false	false	2	2	0

BBB, blood–brain barrier (0 (Very high penetrant); 1 (High); 2 (Medium); 3 (Low); 4 (very low) [30]; Absorption, human intestinal absorption (acceptable range: range is 0–2, where 0 is a good absorption) [28]; Aqueous solubility, (acceptable range: range is 0–3, where 3 is a good solubility) [31]; Cytochrome P450 (CYP450) 2D6 inhibition (false—non-inhibitor, true—inhibitor) [28]; PPB, plasma–protein binding (false—does not bind to plasma proteins, true—binds to plasma proteins) [32,33]; Intestinal absorption (IA).

**Table 6:** Molecules to the computational parameters of USFDA rodent carcinogenicity, Ames mutagenicity and skin irritancy.

Molecules	Mouse Female	Rat Female	Ames Mutagenicity	Skin Irritancy
Hydroxychloroquine	Non-Carcinogen	Non-Carcinogen	Mutagen	None
MolPort-009-219-532	Multi-Carcinogen	Non-Carcinogen	Non-Mutagen	None
MolPort-004-996-519	Non-Carcinogen	Single-Carcinogen	Non-Mutagen	None
MolPort-005-060-605	Non-Carcinogen	Non-Carcinogen	Non-Mutagen	None
MolPort-005-028-274	Non-Carcinogen	Multi-Carcinogen	Non-Mutagen	Mild
MolPort-004-042-669	Non-Carcinogen	Non-Carcinogen	Non-Mutagen	None
MolPort-007-913-111	Multi-Carcinogen	Single-Carcinogen	Non-Mutagen	Mild
MolPort-002-693-933	Multi-Carcinogen	Single-Carcinogen	Non-Mutagen	Mild
MolPort-005-083-430	Non-Carcinogen	Non-Carcinogen	Non-Mutagen	None
MolPort-009-499-144	Non-Carcinogen	Non-Carcinogen	Mutagen	None

**Table 7:** Compliance of molecules with computational toxicity risk parameters.

Molecules	Rate Oral LD_50_ (g/kg Body Weight)	Daphnia EC_50_ (mg/L) *	Rat Chronic LOAEL (g/kg Body Weight)	Fathead Minnow LC_50_ (g/L)
Hydroxychloroquine	0.207	34.619	0.033	0.0240
MolPort-009-219-532	0.520	0.011	0.014	0.0006
MolPort-004-996-519	0.867	0.394	0.005	0.0010
MolPort-005-060-605	4.923	0.104	0.005	0.0004
MolPort-005-028-274	5.528	0.370	0.021	0.0010
MolPort-004-042-669	0.819	1.157	0.024	0.0004
MolPort-007-913-111	1.803	0.022	0.051	0.0003
MolPort-002-693-933	1.560	0.442	0.066	0.0002
MolPort-005-083-430	0.063	0.720	0.014	0.0001
MolPort-009-499-144	1.065	2.801	0.016	0.0020

* Daphnia EC_50_—the effect concentration of a substance that causes adverse effects on 50% of the test population *Daphnia magna*; Rat chronic —Lowest observed adverse effect level (LOAEL); Fathead minnow—Short-term toxicity to fish.

**Table 8:** Carcinogenic potency—tolerated dose (TD_50_, mg/kg body weight/day).

Molecules	Mouse	Rat	RMTD *
Hydroxychloroquine	13.868	1.305	357
MolPort-009-219-532	147.089	51.500	90
MolPort-004-996-519	43.816	1.234	83
MolPort-005-060-605	3.365	0.445	26
MolPort-005-028-274	329.611	25.088	89
MolPort-004-042-669	178.986	9.745	26
MolPort-007-913-111	116.065	11.490	76
MolPort-002-693-933	80.973	10.346	91
MolPort-005-083-430	8.858	56.407	44
MolPort-009-499-144	496.259	15.599	42

* Rat Maximum Tolerated Dose (mg/kg body weight).

**Table 9:** Prediction of biological activity of promising molecules.

Molecule	Molinspiration		PASS		
	Score	Bioactivity	Pa [a]	Pi [b]	Biological Activity
11b	0.65 0.28	Protease inhibitor Enzyme inhibitor	0.265	0.016	Protease inhibitor
MolPort-009-219-532	0.11 0.04	Protease inhibitor Enzyme inhibitor	-	-	-
MolPort-004-996-519	−0.08 −0.17	Protease inhibitor Enzyme inhibitor	-	-	-
MolPort-005-060-605	−0.48 −0.35	Protease inhibitor Enzyme inhibitor	-	-	-
MolPort-005-028-274	−0.36 −0.47	Protease inhibitor Enzyme inhibitor	0.134	0.059	Protease inhibitor
MolPort-009-499-144	−0.52 −0.47	Protease inhibitor Enzyme inhibitor	-	-	-

[a] Pa (probability to be active); [b] Pi (probability to be inactive).

**Table 10:** Prediction of Synthetic Accessibility (SA) of selected molecules.

Molecules	SA	Target
MolPort-007-913-111	65.579	ACE2
MolPort-002-693-933	79.254	
MolPort-004-042-669	67.940	
MolPort-005-131-430	61.351	
MolPort-005-060-605	67.338	
MolPort-009-219-532	81.768	Mpro
MolPort-004-996-519	68.009	
MolPort-005-028-274	67.051	
MolPort-009-499-144	76.392	

**Table 11:** Prediction of lipophilicity through the free web tool SwissADME *.

Moleclues	iLOGP	XLOGP	WLOGP	MLOGP	SILICOS-IT	Consensus LogP
Pivot	3.58	3.58	3.59	2.35	3.73	3.37
MolPort-007-913-111	4.50	4.10	4.13	2.71	5.60	4.21
MolPort-002-693-933	3.33	4.12	3.79	3.46	5.20	3.98
MolPort-004-042-669	3.99	3.55	4.05	2.03	4.62	3.65
MolPort-005-131-430	4.17	4.02	3.23	2.78	4.48	3.74
MolPort-005-060-605	4.61	4.67	4.90	2.76	5.71	4.53
MolPort-009-219-532	4.58	4.51	4.37	2.98	4.78	4.24
MolPort-004-996-519	4.47	4.07	4.48	3.28	4.99	4.26
MolPort-005-028-274	3.46	3.18	3.76	3.23	5.62	3.85
MolPort-009-499-144	2.95	3.04	5.09	1.56	5.05	3.54

* iLOGP: physics-based method relying on free energies of solvation in n-octanol and water calculated by the Generalized-Born and solvent accessible surface area model; XLOGP: an atomistic method including corrective factors and knowledge-based library; WLOGP, implementation of a purely atomistic method based on the fragmental system of Wildman and Crippen; MLOGP: an archetype of a topological method relying on a linear relationship with 13 molecular descriptors; SILICOS-IT: a hybrid method relying on 27 fragments and 7 topological descriptors.

**Table 12:** Prediction of solubility through the free web tool SwissADME.

Moleclues	ESOL	Ali	SILICOS-IT	Consensus LogS
Pivot	−3.91	−4.28	−6.35	−5.81
MolPort-007-913-111	−4.69	−5.73	−5.99	−5.95
MolPort-002-693-933	−4.29	−4.89	−7.10	−5.35
MolPort-004-042-669	−4.35	−5.17	−5.67	−6.33
MolPort-005-131-430	−4.63	−4.66	−7.43	−6.03
MolPort-005-060-605	−5.01	−5.40	−7.02	−5.42
MolPort-009-219-532	−4.57	−4.88	−6.97	−5.27
MolPort-004-996-519	−4.68	−4.84	−7.45	−5.94
MolPort-005-028-274	−4.06	−4.27	−8.57	−6.58
MolPort-009-499-144	−3.92	−4.39	−6.72	−6.57

## Data Availability

Not applicable.

## Supplementary Materials

The following supporting information can be downloaded at: https://www.mdpi.com/article/10.3390/ijms23031781/s1.
