# Supplementary material for: Identification of Potential Antiviral Inhibitors from Hydroxychloroquine and 1,2,4,5-Tetraoxanes Analogues and Investigation of the Mechanism of Action in SARS-CoV-2
Source: Int J Mol Sci. 2022 Feb 4;23(3):1781. doi: 10.3390/ijms23031781 (PMC8836247; doi:10.3390/ijms23031781)
Supplement: Supplementary file 1 [file ijms-23-01781-s001.zip › ijms-1547352 supplementary materials final.pdf]

International Journal of
Molecular Sciences

MDPI

Supplementary Materials

Figure S1. Two-dimensional (2D) structures of molecules set.
Table S1. Computational parameters of oral bioavailability following the Lipinski rule (R5) for the molecules.
Table S2. Computational pharmacokinetic parameters (ADME) of the structures.
Table S3. Computational parameters of USFDA rodent carcinogenicity. Ames mutagenicity. developmental toxicity potential. aerobic biodegradability. ocular irritancy and skin irritancy.
Table S4. Compliance of molecules with computational toxicity risk parameters.
Table S5. Binding affinity values of ligands at ACE2 and M^{pro} receptors.
Table S6. Prediction of Synthetic Accessibility (SA) of the ligands

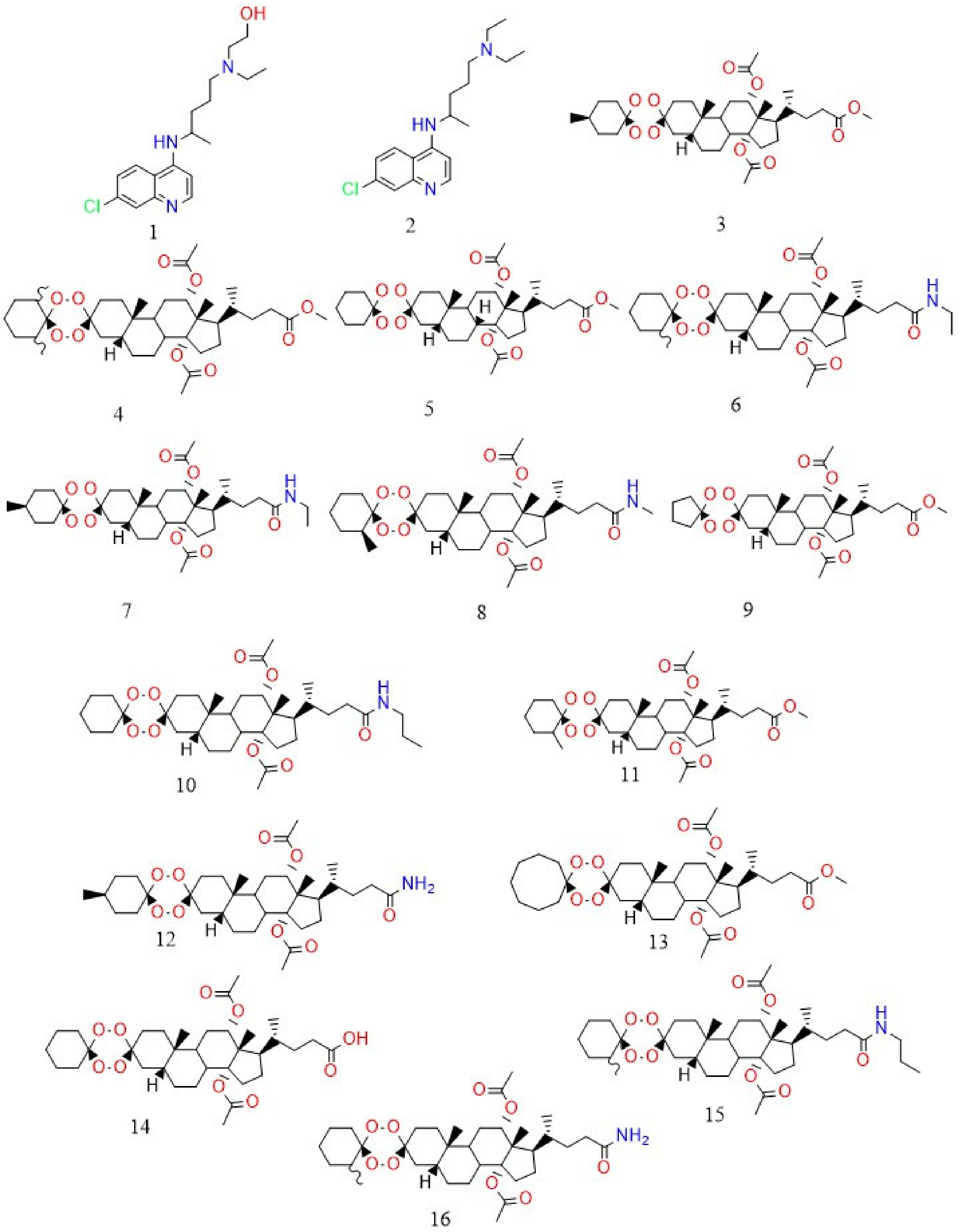

Figure S1. Two-dimensional (2D) structures of molecules set.

Table S1. Computational parameters of oral bioavailability following the Lipinski rule (R5) for the molecules.

Molecules	Oral Bioavailability	MW	Alog P	HBD	HBA	R5
Normal range	(<140 A°^{2})	(<500)	(≤5)	(≤5)	(≤10)	Max 4
Hydroxychloroquine	48.239	335.872	3.457	2	4	0
Chloroquine	27.423	319.872	4.345	1	3	0
MolPort-009-219-532	30.142	355.471	4.755	0	4	0
MolPort-045-904-593	59.791	392.877	3.388	1	6	0
MolPort-006-669-322	69.152	338.485	3.250	2	3	0
MolPort-010-847-948	88.170	420.522	3.110	1	5	0
MolPort-005-109-508	53.126	423.548	4.042	0	5	0
MolPort-009-624-542	73.642	457.178	3.882	1	4	0
MolPort-006-518-079	79.240	424.414	4.012	1	4	0
MolPort-007-760-271	68.395	440.578	3.413	1	4	0
MolPort-004-999-716	51.323	484.626	3.919	1	5	0
MolPort-006-669-183	76.995	448.619	3.550	1	4	0
MolPort-005-116-387	53.958	374.474	3.285	0	5	0
MolPort-006-521-801	78.463	460.95	3.950	0	5	0
MolPort-006-669-319	73.642	399.568	3.682	1	4	0
MolPort-005-052-112	55.203	415.956	3.815	2	4	0
MolPort-009-089-697	69.152	388.888	3.877	2	3	0
MolPort-028-816-982	65.272	417.514	3.995	1	4	0
MolPort-007-913-109	54.676	405.554	4.381	1	5	0
MolPort-005-026-316	69.152	452.561	3.821	2	3	0
MolPort-007-913-113	81.434	433.564	4.294	2	5	0
MolPort-002-297-028	51.485	442.774	4.723	2	4	0
MolPort-004-063-477	61.951	380.819	4.353	0	6	0
MolPort-005-126-472	45.027	358.474	3.296	0	4	0
MolPort-002-604-292	61.391	364.82	4.456	0	5	0
MolPort-004-996-519	51.323	414.513	4.588	1	4	0
MolPort-009-239-957	58.766	430.56	4.890	0	6	0
MolPort-006-669-332	56.341	337.497	4.075	1	3	0
MolPort-005-060-605	45.027	398.538	4.677	0	4	0
MolPort-005-028-274	69.152	416.461	3.347	2	3	0
MolPort-044-400-535	68.224	384.469	3.866	1	5	0
MolPort-005-695-540	51.323	447.365	3.482	1	4	0
MolPort-039-018-869	65.357	413.576	4.848	2	4	0
MolPort-002-714-306	60.986	372.481	3.974	0	6	0
MolPort-007-913-256	81.434	433.564	4.292	2	5	0
MolPort-003-330-718	90.501	437.576	2.936	1	6	0
MolPort-000-848-037	71.052	436.543	3.773	1	5	0
MolPort-005-008-705	68.624	410.506	4.338	1	4	0
MolPort-004-042-669	66.740	417.518	3.416	0	6	0
MolPort-007-913-111	54.676	419.581	4.826	1	5	0
MolPort-007-913-243	81.434	419.538	3.973	2	5	0
MolPort-002-693-933	50.364	324.417	4.586	1	3	0

MolPort-005-083-430	40.152	426.618	4.778	0	5	0
MolPort-002-240-450	64.744	405.508	4.338	0	7	0
MolPort-010-232-623	59.166	486.548	4.037	0	5	0
MolPort-028-787-272	59.694	342.497	2.944	1	4	0
MolPort-002-513-970	72.410	403.555	4.247	2	4	0
MolPort-007-913-196	81.434	419.538	3.838	2	5	0
MolPort-005-131-430	45.746	423.591	4.054	1	4	0
MolPort-001-683-506	61.391	366.836	4.978	0	5	0
MolPort-044-684-282	63.574	399.569	4.505	2	3	0
MolPort-009-499-144	80.327	398.376	3.166	2	4	0
MolPort-010-813-978	92.050	405.511	2.548	2	4	0
MolPort-005-002-644	60.253	494.665	4.586	1	5	0
MolPort-009-511-439	84.227	498.562	3.494	2	5	0
MolPort-009-647-594	79.240	424.533	3.662	1	4	0

Table S2. Computational pharmacokinetic parameters (ADME) of the structures.

Molecules	PPB	Hepatotoxicidade	CYP2D6 Binding	Solubility	BBB	IA
Hydroxychloroquine	false (poorly bounded)	true (toxic)	true (inhibitor)	3 (good)	1 (good)	0 (good)
Chloroquine	true (highly bounded)	true (toxic)	true (inhibitor)	2 (low)	0 (very good)	0 (good)
MolPort-009-219-532	true (highly bounded)	false (non-toxic)	true (inhibitor)	2 (low)	0 (very good)	0 (good)
MolPort-045-904-593	true (highly bounded)	false (non-toxic)	true (inhibitor)	2 (low)	2 (medium)	0 (good)
MolPort-006-669-322	true (highly bounded)	false (non-toxic)	false (non-inhibitor)	3 (good)	2 (medium)	0 (good)
MolPort-010-847-948	true (highly bounded)	false (non-toxic)	false (non-inhibitor)	2 (low)	3 (low)	0 (good)
MolPort-005-109-508	true (highly bounded)	false (non-toxic)	false (non-inhibitor)	2 (low)	1 (good)	0 (good)
MolPort-009-624-542	true (highly bounded)	false (non-toxic)	false (non-inhibitor)	2 (low)	2 (medium)	0 (good)
MolPort-006-518-079	true (highly bounded)	false (non-toxic)	false (non-inhibitor)	2 (low)	2 (medium)	0 (good)
MolPort-007-760-271	true (highly bounded)	false (non-toxic)	false (non-inhibitor)	2 (low)	2 (medium)	0 (good)
MolPort-004-999-716	false (poorly bounded)	false (non-toxic)	false (non-inhibitor)	2 (low)	1 (good)	0 (good)
MolPort-006-669-183	true (highly bounded)	false (non-toxic)	false (non-inhibitor)	2 (low)	2 (medium)	0 (good)
MolPort-005-116-387	true (highly bounded)	false (non-toxic)	false (non-inhibitor)	2 (low)	1 (good)	0 (good)
MolPort-006-521-801	true (highly bounded)	false (non-toxic)	false (non-inhibitor)	2 (low)	2 (medium)	0 (good)
MolPort-006-669-319	true (highly bounded)	false (non-toxic)	false (non-inhibitor)	2 (low)	2 (medium)	0 (good)
MolPort-005-052-112	true (highly bounded)	false (non-toxic)	false (non-inhibitor)	2 (low)	1 (good)	0 (good)
MolPort-009-089-697	true (highly bounded)	false (non-toxic)	false (non-inhibitor)	2 (low)	2 (medium)	0 (good)
MolPort-028-816-982	true (highly bounded)	false (non-toxic)	false (non-inhibitor)	2 (low)	1 (good)	0 (good)
MolPort-007-913-109	true (highly bounded)	false (non-toxic)	false (non-inhibitor)	2 (low)	1 (good)	0 (good)
MolPort-005-026-316	true (highly bounded)	false (non-toxic)	false (non-inhibitor)	2 (low)	2 (medium)	0 (good)
MolPort-007-913-113	false (poorly bounded)	false (non-toxic)	false (non-inhibitor)	2 (low)	2 (medium)	0 (good)
MolPort-002-297-028	true (highly bounded)	false (non-toxic)	true (inhibitor)	2 (low)	1 (good)	0 (good)
MolPort-004-063-477	true (highly bounded)	false (non-toxic)	false (non-inhibitor)	2 (low)	1 (good)	0 (good)
MolPort-005-126-472	true (highly bounded)	false (non-toxic)	true (inhibitor)	2 (low)	1 (good)	0 (good)
MolPort-002-604-292	true (highly bounded)	false (non-toxic)	false (non-inhibitor)	2 (low)	1 (good)	0 (good)
MolPort-004-996-519	true (highly bounded)	false (non-toxic)	false (non-inhibitor)	2 (low)	1 (good)	0 (good)
MolPort-009-239-957	true (highly bounded)	false (non-toxic)	false (non-inhibitor)	2 (low)	1 (good)	0 (good)
MolPort-006-669-332	true (highly bounded)	false (non-toxic)	false (non-inhibitor)	2 (low)	1 (good)	0 (good)
MolPort-005-060-605	true (highly bounded)	false (non-toxic)	false (non-inhibitor)	2 (low)	1 (good)	0 (good)
MolPort-005-028-274	true (highly bounded)	false (non-toxic)	false (non-inhibitor)	2 (low)	2 (medium)	0 (good)
MolPort-044-400-535	true (highly bounded)	false (non-toxic)	false (non-inhibitor)	2 (low)	2 (medium)	0 (good)
MolPort-005-695-540	true (highly bounded)	false (non-toxic)	false (non-inhibitor)	2 (low)	1 (good)	0 (good)
MolPort-039-018-869	true (highly bounded)	false (non-toxic)	false (non-inhibitor)	1 (poor)	1 (good)	0 (good)
MolPort-002-714-306	true (highly bounded)	false (non-toxic)	false (non-inhibitor)	2 (low)	1 (good)	0 (good)
MolPort-007-913-256	true (highly bounded)	false (non-toxic)	false (non-inhibitor)	2 (low)	2 (medium)	0 (good)
MolPort-003-330-718	true (highly bounded)	false (non-toxic)	false (non-inhibitor)	2 (low)	3 (low)	0 (good)
MolPort-000-848-037	true (highly bounded)	false (non-toxic)	false (non-inhibitor)	2 (low)	2 (medium)	0 (good)
MolPort-005-008-705	true (highly bounded)	false (non-toxic)	false (non-inhibitor)	2 (low)	1 (good)	0 (good)
MolPort-004-042-669	true (highly bounded)	false (non-toxic)	false (non-inhibitor)	2 (low)	2 (medium)	0 (good)
MolPort-007-913-111	false (poorly bounded)	false (non-toxic)	false (non-inhibitor)	2 (low)	1 (good)	0 (good)
MolPort-007-913-243	true (highly bounded)	false (non-toxic)	false (non-inhibitor)	2 (low)	2 (medium)	0 (good)
MolPort-002-693-933	true (highly bounded)	false (non-toxic)	false (non-inhibitor)	2 (low)	1 (good)	0 (good)
MolPort-005-083-430	true (highly bounded)	false (non-toxic)	true (inhibitor)	2 (low)	1 (good)	0 (good)

MolPort-002-240-450	true (highly bounded)	false (non-toxic)	false (non-inhibitor)	2 (low)	1 (good)	0 (good)
MolPort-010-232-623	true (highly bounded)	false (non-toxic)	false (non-inhibitor)	2 (low)	1 (good)	0 (good)
MolPort-028-787-272	true (highly bounded)	false (non-toxic)	false (non-inhibitor)	3 (good)	2 (medium)	0 (good)
MolPort-002-513-970	true (highly bounded)	false (non-toxic)	false (non-inhibitor)	2 (low)	1 (good)	0 (good)
MolPort-007-913-196	false (poorly bounded)	false (non-toxic)	false (non-inhibitor)	2 (low)	2 (medium)	0 (good)
MolPort-005-131-430	true (highly bounded)	false (non-toxic)	false (non-inhibitor)	2 (low)	1 (good)	0 (good)
MolPort-001-683-506	true (highly bounded)	false (non-toxic)	false (non-inhibitor)	2 (low)	1 (good)	0 (good)
MolPort-044-684-282	true (highly bounded)	false (non-toxic)	false (non-inhibitor)	2 (low)	1 (good)	0 (good)
MolPort-009-499-144	true (highly bounded)	false (non-toxic)	false (non-inhibitor)	2 (low)	2 (medium)	0 (good)
MolPort-010-813-978	true (highly bounded)	false (non-toxic)	false (non-inhibitor)	3 (good)	3 (low)	0 (good)
MolPort-005-002-644	false (poorly bounded)	false (non-toxic)	false (non-inhibitor)	2 (low)	1 (good)	0 (good)
MolPort-009-511-439	true (highly bounded)	false (non-toxic)	false (non-inhibitor)	2 (low)	2 (medium)	0 (good)
MolPort-009-647-594	true (highly bounded)	false (non-toxic)	false (non-inhibitor)	2 (low)	2 (medium)	0 (good)

Table S3. Computational parameters of USFDA rodent carcinogenicity. Ames mutagenicity. developmental toxicity potential. aerobic biodegradability. ocular irritancy and skin irritancy.

Molecules	Mouse Female	Mouse Male	Rat Female	Rat Male	Ames Mutagenicity	DTP	Skin Irritancy	AB
Hydroxychloroquine	Non-Carcinogen	Non-Carcinogen	Non-Carcinogen	Non-Carcinogen	Mutagen	Toxic	None	Non-Degradable
Chloroquine	Non-Carcinogen	Non-Carcinogen	Non-Carcinogen	Non-Carcinogen	Mutagen	Non-Toxic	None	Non-Degradable
MolPort-009-219-532	Multi-Carcinogen	Non-Carcinogen	Non-Carcinogen	Non-Carcinogen	Non-Mutagen	Toxic	None	Non-Degradable
MolPort-045-904-593	Non-Carcinogen	Non-Carcinogen	Non-Carcinogen	Non-Carcinogen	Non-Mutagen	Toxic	Mild	Non-Degradable
MolPort-006-669-322	Non-Carcinogen	Multi-Carcinogen	Non-Carcinogen	Multi-Carcinogen	Non-Mutagen	Non-Toxic	Moderate	Degradable
MolPort-010-847-948	Single-Carcinogen	Non-Carcinogen	Non-Carcinogen	Non-Carcinogen	Non-Mutagen	Non-Toxic	Mild	Degradable
MolPort-005-109-508	Non-Carcinogen	Non-Carcinogen	Non-Carcinogen	Non-Carcinogen	Non-Mutagen	Toxic	None	Degradable
MolPort-009-624-542	Non-Carcinogen	Non-Carcinogen	Non-Carcinogen	Non-Carcinogen	Non-Mutagen	Toxic	None	Degradable
MolPort-006-518-079	Non-Carcinogen	Multi-Carcinogen	Non-Carcinogen	Non-Carcinogen	Non-Mutagen	Non-Toxic	None	Non-Degradable
MolPort-007-760-271	Non-Carcinogen	Non-Carcinogen	Non-Carcinogen	Non-Carcinogen	Non-Mutagen	Toxic	None	Degradable
MolPort-004-999-716	Non-Carcinogen	Non-Carcinogen	Non-Carcinogen	Non-Carcinogen	Non-Mutagen	Non-Toxic	None	Degradable
MolPort-006-669-183	Non-Carcinogen	Single-Carcinogen	Single-Carcinogen	Non-Carcinogen	Non-Mutagen	Non-Toxic	Mild	Degradable
MolPort-005-116-387	Non-Carcinogen	Non-Carcinogen	Non-Carcinogen	Non-Carcinogen	Non-Mutagen	Toxic	None	Non-Degradable
MolPort-006-521-801	Non-Carcinogen	Single-Carcinogen	Non-Carcinogen	Non-Carcinogen	Non-Mutagen	Toxic	None	Non-Degradable
MolPort-006-669-319	Non-Carcinogen	Non-Carcinogen	Non-Carcinogen	Single-Carcinogen	Non-Mutagen	Toxic	None	Degradable
MolPort-005-052-112	Non-Carcinogen	Non-Carcinogen	Non-Carcinogen	Non-Carcinogen	Non-Mutagen	Toxic	None	Non-Degradable
MolPort-009-089-697	Multi-Carcinogen	Non-Carcinogen	Non-Carcinogen	Non-Carcinogen	Non-Mutagen	Toxic	None	Non-Degradable
MolPort-028-816-982	Non-Carcinogen	Non-Carcinogen	Non-Carcinogen	Non-Carcinogen	Non-Mutagen	Non-Toxic	None	Non-Degradable
MolPort-007-913-109	Non-Carcinogen	Non-Carcinogen	Non-Carcinogen	Non-Carcinogen	Non-Mutagen	Toxic	None	Non-Degradable
MolPort-005-026-316	Non-Carcinogen	Non-Carcinogen	Single-Carcinogen	Non-Carcinogen	Non-Mutagen	Toxic	Mild	Non-Degradable
MolPort-007-913-113	Non-Carcinogen	Non-Carcinogen	Non-Carcinogen	Non-Carcinogen	Non-Mutagen	Non-Toxic	None	Degradable
MolPort-002-297-028	Non-Carcinogen	Non-Carcinogen	Single-Carcinogen	Non-Carcinogen	Non-Mutagen	Non-Toxic	Mild	Degradable
MolPort-004-063-477	Non-Carcinogen	Non-Carcinogen	Non-Carcinogen	Non-Carcinogen	Non-Mutagen	Toxic	None	Non-Degradable
MolPort-005-126-472	Non-Carcinogen	Multi-Carcinogen	Single-Carcinogen	Non-Carcinogen	Non-Mutagen	Non-Toxic	Moderate	Degradable
MolPort-002-604-292	Non-Carcinogen	Multi-Carcinogen	Non-Carcinogen	Non-Carcinogen	Non-Mutagen	Non-Toxic	Mild	Degradable
MolPort-004-996-519	Non-Carcinogen	Non-Carcinogen	Single-Carcinogen	Non-Carcinogen	Non-Mutagen	Non-Toxic	None	Degradable
MolPort-009-239-957	Non-Carcinogen	Non-Carcinogen	Non-Carcinogen	Non-Carcinogen	Non-Mutagen	Non-Toxic	None	Degradable

MolPort-006-669-332	Non-Carcinogen	Non-Carcinogen	Non-Carcinogen	Non-Carcinogen	Non-Mutagen	Toxic	None	Non-Degradable
MolPort-005-060-605	Non-Carcinogen	Non-Carcinogen	Non-Carcinogen	Non-Carcinogen	Non-Mutagen	Non-Toxic	None	Non-Degradable
MolPort-005-028-274	Non-Carcinogen	Multi-Carcinogen	Multi-Carcinogen	Single-Carcinogen	Non-Mutagen	Toxic	Mild	Non-Degradable
MolPort-044-400-535	Non-Carcinogen	Non-Carcinogen	Non-Carcinogen	Non-Carcinogen	Non-Mutagen	Non-Toxic	None	Non-Degradable
MolPort-005-695-540	Non-Carcinogen	Single-Carcinogen	Non-Carcinogen	Non-Carcinogen	Non-Mutagen	Non-Toxic	None	Non-Degradable
MolPort-039-018-869	Non-Carcinogen	Non-Carcinogen	Single-Carcinogen	Non-Carcinogen	Non-Mutagen	Toxic	None	Non-Degradable
MolPort-002-714-306	Non-Carcinogen	Non-Carcinogen	Non-Carcinogen	Non-Carcinogen	Non-Mutagen	Toxic	None	Degradable
MolPort-007-913-256	Non-Carcinogen	Single-Carcinogen	Single-Carcinogen	Non-Carcinogen	Non-Mutagen	Toxic	None	Degradable
MolPort-003-330-718	Non-Carcinogen	Non-Carcinogen	Non-Carcinogen	Non-Carcinogen	Non-Mutagen	Toxic	Mild	Degradable
MolPort-000-848-037	Non-Carcinogen	Non-Carcinogen	Non-Carcinogen	Non-Carcinogen	Non-Mutagen	Toxic	None	Non-Degradable
MolPort-005-008-705	Non-Carcinogen	Non-Carcinogen	Non-Carcinogen	Non-Carcinogen	Non-Mutagen	Non-Toxic	None	Non-Degradable
MolPort-004-042-669	Non-Carcinogen	Non-Carcinogen	Non-Carcinogen	Non-Carcinogen	Non-Mutagen	Toxic	None	Non-Degradable
MolPort-007-913-111	Multi-Carcinogen	Non-Carcinogen	Single-Carcinogen	Non-Carcinogen	Non-Mutagen	Non-Toxic	Mild	Degradable
MolPort-007-913-243	Non-Carcinogen	Non-Carcinogen	Non-Carcinogen	Non-Carcinogen	Non-Mutagen	Toxic	Mild	Non-Degradable
MolPort-002-693-933	Multi-Carcinogen	Multi-Carcinogen	Single-Carcinogen	Single-Carcinogen	Non-Mutagen	Toxic	Mild	Degradable
MolPort-005-083-430	Non-Carcinogen	Non-Carcinogen	Non-Carcinogen	Non-Carcinogen	Non-Mutagen	Non-Toxic	None	Non-Degradable
MolPort-002-240-450	Non-Carcinogen	Non-Carcinogen	Non-Carcinogen	Non-Carcinogen	Non-Mutagen	Non-Toxic	Mild	Non-Degradable
MolPort-010-232-623	Non-Carcinogen	Non-Carcinogen	Single-Carcinogen	Single-Carcinogen	Non-Mutagen	Non-Toxic	Mild	Degradable
MolPort-028-787-272	Non-Carcinogen	Single-Carcinogen	Non-Carcinogen	Non-Carcinogen	Non-Mutagen	Toxic	None	Degradable
MolPort-002-513-970	Non-Carcinogen	Non-Carcinogen	Non-Carcinogen	Non-Carcinogen	Non-Mutagen	Non-Toxic	None	Non-Degradable
MolPort-007-913-196	Non-Carcinogen	Non-Carcinogen	Non-Carcinogen	Non-Carcinogen	Non-Mutagen	Non-Toxic	None	Non-Degradable
MolPort-005-131-430	Non-Carcinogen	Non-Carcinogen	Non-Carcinogen	Non-Carcinogen	Non-Mutagen	Toxic	None	Non-Degradable
MolPort-001-683-506	Non-Carcinogen	Non-Carcinogen	Non-Carcinogen	Non-Carcinogen	Non-Mutagen	Non-Toxic	None	Non-Degradable
MolPort-044-684-282	Non-Carcinogen	Non-Carcinogen	Non-Carcinogen	Non-Carcinogen	Non-Mutagen	Non-Toxic	Mild	Degradable
MolPort-009-499-144	Non-Carcinogen	Non-Carcinogen	Non-Carcinogen	Non-Carcinogen	Mutagen	Toxic	None	Non-Degradable
MolPort-010-813-978	Non-Carcinogen	Non-Carcinogen	Non-Carcinogen	Non-Carcinogen	Mutagen	Non-Toxic	None	Non-Degradable
MolPort-005-002-644	Multi-Carcinogen	Non-Carcinogen	Non-Carcinogen	Non-Carcinogen	Non-Mutagen	Toxic	None	Non-Degradable
MolPort-009-511-439	Non-Carcinogen	Non-Carcinogen	Non-Carcinogen	Non-Carcinogen	Non-Mutagen	Toxic	Mild	Non-Degradable
MolPort-009-647-594	Non-Carcinogen	Multi-Carcinogen	Non-Carcinogen	Multi-Carcinogen	Non-Mutagen	Non-Toxic	Moderate	Degradable

Table S4. Compliance of molecules with computational toxicity risk parameters.

Molecules	Rate Oral LD_{50} (g/kg Body Weight)	Daphnia EC_{50} (mg/L)	Rat Chronic LOAEL (g/kg Body Weight)	Fathead Minnow LC_{50} (g/L)
Hydroxychloroquine	0.206628	34.6185	0.0329227	0.0240234
Chloroquine	0.15613	2.82639	0.0241049	0.00638702
MolPort-009-219-532	0.519074	0.0113327	0.0143892	0.000647581
MolPort-045-904-593	1.63109	0.480538	0.00612089	0.000682701
MolPort-006-669-322	2.53059	0.578267	0.00754108	0.000287506
MolPort-010-847-948	1.50569	1.64518	0.0230496	0.00283626
MolPort-005-109-508	2.34626	0.55976	0.00169879	1.93741e-05
MolPort-009-624-542	7.96501	0.0686546	0.0379408	6.09624e-05
MolPort-006-518-079	0.4479	0.743687	0.00828422	0.000317579
MolPort-007-760-271	1.05304	0.694215	0.00751075	0.000890254
MolPort-004-999-716	0.215143	0.136903	0.00160099	6.72694e-05
MolPort-006-669-183	1.66476	0.814212	0.00664927	2.29034e-05
MolPort-005-116-387	1.21449	0.533122	0.0191592	0.00710619
MolPort-006-521-801	1.18007	1.43138	0.0114201	0.000521094
MolPort-006-669-319	6.67192	0.911401	0.00503748	3.57542e-05
MolPort-005-052-112	1.55741	0.937148	0.0131351	0.00548019
MolPort-009-089-697	4.62639	2.19233	0.0595124	0.00338829
MolPort-028-816-982	0.344187	0.434683	0.00352219	0.000933634
MolPort-007-913-109	1.66563	0.0503399	0.0550836	0.000698277
MolPort-005-026-316	0.939254	0.38515	0.0318489	0.000962445
MolPort-007-913-113	7.64343	0.150338	0.0426269	0.000189959
MolPort-002-297-028	10.5937	0.208959	0.0888331	0.0011936
MolPort-004-063-477	3.00361	0.186291	0.0168658	0.00032414
MolPort-005-126-472	2.73546	0.470462	0.0188029	0.00732731
MolPort-002-604-292	4.22948	0.391261	0.0418871	8.11418e-05
MolPort-004-996-519	0.866938	0.394266	0.00450013	0.000517998
MolPort-009-239-957	8.37807	0.0321625	0.00705534	2.06246e-05
MolPort-006-669-332	4.56936	0.694498	0.00562308	0.000224901
MolPort-005-060-605	4.92316	0.103711	0.00508813	0.000404192
MolPort-005-028-274	5.52822	0.370371	0.0209951	0.000572707
MolPort-044-400-535	1.39146	0.590401	0.0250407	0.00143846
MolPort-005-695-540	1.02577	0.84207	0.0178195	0.00177727
MolPort-039-018-869	0.115168	0.131652	0.0027505	0.000563123
MolPort-002-714-306	4.97782	0.307299	0.085262	0.00116953
MolPort-007-913-256	5.21469	0.282713	0.0450132	0.000107131
MolPort-003-330-718	0.208377	0.170916	0.0179479	0.000560762
MolPort-000-848-037	1.16646	2.95073	0.078792	0.00222931
MolPort-005-008-705	0.880832	0.186796	0.00764375	0.0011703
MolPort-004-042-669	0.818649	1.15746	0.0243816	0.000426359
MolPort-007-913-111	1.80343	0.021842	0.0514566	0.000327461
MolPort-007-913-243	5.22694	0.284565	0.0374126	0.000354254
MolPort-002-693-933	1.55997	0.441939	0.0663394	0.000233932
MolPort-005-083-430	0.0634634	0.719656	0.0137068	0.000147288

MolPort-002-240-450	0.780211	0.0749317	0.00657294	5.79877e-05
MolPort-010-232-623	0.153435	1.63212	0.00769818	0.000178307
MolPort-028-787-272	1.42423	0.753566	0.0498866	0.00776433
MolPort-002-513-970	2.11162	0.919901	0.00532493	0.000313351
MolPort-007-913-196	8.95623	0.227159	0.0397907	0.000360996
MolPort-005-131-430	1.22613	0.819688	0.0146579	0.00196697
MolPort-001-683-506	7.24965	0.132511	0.09593	0.000176762
MolPort-044-684-282	4.42066	8.57627	0.0797648	0.00395037
MolPort-009-499-144	1.06527	2.80075	0.0157899	0.00206903
MolPort-010-813-978	3.40067	1.07902	0.0757667	0.0134553
MolPort-005-002-644	0.923189	1.38632	0.00629606	0.000466151
MolPort-009-511-439	6.92456	6.12515	0.0316054	0.000942829
MolPort-009-647-594	0.243958	1.60554	0.105641	0.000273276

Table S4. (*cont.*) Compliance of molecules with computational toxicity risk parameters.

Carcinogenic Potency TD_{50} (mg/kg Body Weight/Day)			
**Molecules**	**Mouse**	**Rat**	**Rat Maximum Tolerated Dose (g/kg Body Weight)**
Hydroxychloroquine	13.8683	1.30464	0.357402
Chloroquine	9.37816	0.376991	0.198368
MolPort-009-219-532	147.089	51.5002	0.0895825
MolPort-045-904-593	7.35069	1.12958	0.0477912
MolPort-006-669-322	148.593	9.39412	0.0768844
MolPort-010-847-948	70.9216	16.0969	0.0757447
MolPort-005-109-508	3.57508	1.90891	0.0344346
MolPort-009-624-542	457.629	34.106	0.0345973
MolPort-006-518-079	75.1211	20.3393	0.0602477
MolPort-007-760-271	59.6433	1.62658	0.0231931
MolPort-004-999-716	40.3006	0.869267	0.0332906
MolPort-006-669-183	57.8641	31.0552	0.0197732
MolPort-005-116-387	27.1752	1.17545	0.0282444
MolPort-006-521-801	23.4828	2.96423	0.0367389
MolPort-006-669-319	342.56	912.033	0.0354822
MolPort-005-052-112	96.6493	0.0346954	0.0842258
MolPort-009-089-697	208.184	42.6662	0.148747
MolPort-028-816-982	18.2215	0.208099	0.0371243
MolPort-007-913-109	130.609	11.4046	0.0719888
MolPort-005-026-316	134.391	22.0456	0.0718077
MolPort-007-913-113	128.296	5.9438	0.0726094
MolPort-002-297-028	228.499	70.4747	0.128577
MolPort-004-063-477	75.5015	4.24665	0.0577177
MolPort-005-126-472	81.1056	2.87661	0.0254416
MolPort-002-604-292	260.873	26.4585	0.0605204
MolPort-004-996-519	43.8161	1.23396	0.0827132
MolPort-009-239-957	18.9886	24.2648	0.0275296
MolPort-006-669-332	258.535	209.59	0.0634369

MolPort-005-060-605	3.36456	0.444702	0.0261636
MolPort-005-028-274	329.611	25.0882	0.0888492
MolPort-044-400-535	171.785	9.65002	0.0721541
MolPort-005-695-540	94.0308	0.13559	0.0396155
MolPort-039-018-869	1.48708	0.0176144	0.0500809
MolPort-002-714-306	83.2817	12.4719	0.0284433
MolPort-007-913-256	101.882	4.9379	0.065963
MolPort-003-330-718	6.3469	23.2406	0.0370255
MolPort-000-848-037	76.4916	27.4908	0.159796
MolPort-005-008-705	95.0984	16.4368	0.0339939
MolPort-004-042-669	178.986	9.74467	0.0257443
MolPort-007-913-111	116.065	11.4898	0.0763168
MolPort-007-913-243	113.128	4.53826	0.070089
MolPort-002-693-933	80.9734	10.3455	0.0905694
MolPort-005-083-430	8.85774	56.407	0.0435164
MolPort-002-240-450	165.814	104.529	0.0470502
MolPort-010-232-623	31.8064	1.23874	0.0156361
MolPort-028-787-272	135.568	23.9449	0.0907596
MolPort-002-513-970	36.8187	15.9221	0.0713243
MolPort-007-913-196	146.94	6.39101	0.0766533
MolPort-005-131-430	57.0133	0.0283448	0.0430569
MolPort-001-683-506	388.024	27.0801	0.131733
MolPort-044-684-282	51.1957	43.944	0.0884948
MolPort-009-499-144	496.259	15.5986	0.041821
MolPort-010-813-978	196.116	3.49483	0.0509963
MolPort-005-002-644	61.2674	0.309164	0.0239698
MolPort-009-511-439	138.172	51.058	0.0556744
MolPort-009-647-594	284.682	33.2544	0.0671164

Table S5. Binding affinity values of ligands at ACE2 and M^{pro} receptors.

Ligand	Binding Affinity (Kcal/mol)	
	**ACE2**	**M^{pro}**
Hydroxychloroquine	-7.755	-8.337
Chloroquine	-7.709	-7.158
Lopinavir	-7.866	-9.680
Ritonavir	-8.993	-9.594
11b	-8.441	-8.587
MolPort-009-219-532	-7.791	-9.012
MolPort-045-904-593	-7.352	-8.527
MolPort-006-669-322	-7.370	-7.972
MolPort-010-847-948	-7.610	-8.812
MolPort-005-109-508	-8.031	-8.235
MolPort-009-624-542	-7.717	-8.001
MolPort-006-518-079	-7.574	-8.451
MolPort-007-760-271	-7.952	-8.326
MolPort-004-999-716	-7.802	-7.588
MolPort-006-669-183	-7.974	-8.157
MolPort-005-116-387	-7.991	-8.842
MolPort-006-521-801	-8.147	-8.985
MolPort-006-669-319	-6.969	-8.283
MolPort-005-052-112	-7.655	-8.528
MolPort-009-089-697	-8.421	-7.894
MolPort-028-816-982	-8.111	-9.202
MolPort-007-913-109	-7.953	-7.936
MolPort-005-026-316	-8.213	-7.830
MolPort-007-913-113	-7.601	-8.147
MolPort-002-297-028	-8.116	-8.134
MolPort-004-063-477	-7.889	-8.515
MolPort-005-126-472	-7.062	-8.197
MolPort-002-604-292	-7.882	-8.116
MolPort-004-996-519	-8.033	-9.008
MolPort-009-239-957	-7.953	-8.963
MolPort-006-669-332	-7.574	-8.297
MolPort-005-060-605	-8.138	-9.076
MolPort-005-028-274	-8.116	-8.883
MolPort-044-400-535	-8.090	-8.552
MolPort-005-695-540	-7.847	-8.803
MolPort-039-018-869	-7.998	-8.247
MolPort-002-714-306	-7.853	-8.502
MolPort-007-913-256	-7.911	-8.786
MolPort-003-330-718	-7.833	-8.132
MolPort-000-848-037	-7.858	-8.099
MolPort-005-008-705	-8.197	-8.638

MolPort-004-042-669	-8.228	-8.647
MolPort-007-913-111	-8.540	-8.016
MolPort-007-913-243	-8.367	-8.229
MolPort-002-693-933	-8.440	-8.280
MolPort-005-083-430	-8.263	-8.874
MolPort-002-240-450	-7.662	-8.441
MolPort-010-232-623	-8.236	-8.372
MolPort-028-787-272	-7.496	-7.641
MolPort-002-513-970	-7.799	-8.631
MolPort-007-913-196	-8.252	-8.156
MolPort-005-131-430	-8.226	-8.395
MolPort-001-683-506	-8.218	-8.727
MolPort-044-684-282	-8.036	-8.637
MolPort-009-499-144	-7.676	-8.818
MolPort-010-813-978	-7.472	-8.567
MolPort-005-002-644	-8.112	-8.815
MolPort-009-511-439	-7.420	-7.983
MolPort-009-647-594	-7.346	-8.555

Table S6. Prediction of Synthetic Accessibility (SA) of the ligands.

Ligand	SA
Hydroxychloroquine	69.792
Chloroquine	70.325
Lopinavir	36.612
Ritonavir	32.899
11b	47.747
MolPort-009-219-532	81.768
MolPort-010-847-948	68.495
MolPort-005-116-387	71.440
MolPort-006-521-801	66.419
MolPort-009-089-697	71.324
MolPort-028-816-982	32.472
MolPort-005-026-316	56.301
MolPort-002-297-028	77.504
MolPort-004-996-519	68.009
MolPort-009-239-957	63.779
MolPort-005-060-605	67.338
MolPort-005-028-274	67.051
MolPort-044-400-535	74.829
MolPort-005-695-540	76.412
MolPort-007-913-256	57.312
MolPort-005-008-705	68.168
MolPort-004-042-669	67.940
MolPort-007-913-111	65.579
MolPort-007-913-243	71.178
MolPort-002-693-933	79.254
MolPort-005-083-430	59.789
MolPort-010-232-623	70.187
MolPort-002-513-970	45.605
MolPort-007-913-196	70.662
MolPort-005-131-430	61.351
MolPort-001-683-506	73.593
MolPort-044-684-282	74.706
MolPort-009-499-144	76.392
MolPort-005-002-644	50.303
MolPort-009-647-594	67.407
